# On‐Water Surface Synthesis of 2D Conjugated Metal–Organic Framework Films With Controllable Layer Orientation Enabling High‐Performance Chemiresistive Sensing

**DOI:** 10.1002/adma.73785

**Published:** 2026-06-23

**Authors:** Jianjun Zhang, Víctor García‐López, Yutong Wu, Shuai Fu, Yucong Chen, Arsh S. Hazrah, Petko Petkov, Mike Hambsch, Feng Ni, Alina Müller, Ye Yang, Leif Riemenschneider, Shirong Huang, Albertus Mattheüs Sandee, Yilv Guo, David Silva‐Brea, Jan‐Ole Joswig, Stefan C. B. Mannsfeld, Thomas Heine, Gianaurelio Cuniberti, Mischa Bonn, Zhiyong Wang, Xinliang Feng

**Affiliations:** ^1^ Max Planck Institute of Microstructure Physics Halle Germany; ^2^ Faculty of Chemistry and Food Chemistry & Center for Advanced Electronics Dresden (CFAED) TUD Dresden University of Technology Dresden Germany; ^3^ Institute for Materials Science and Max Bergmann Center for Biomaterials TUD Dresden University of Technology Dresden Germany; ^4^ Max Planck Institute for Polymer Research Mainz Germany; ^5^ Faculty of Chemistry and Pharmacy University of Sofia Sofia Bulgaria; ^6^ Faculty of Electrical and Computer Engineering and Center for Advancing Electronics Dresden (CFAED) TUD Dresden University of Technology Dresden Germany; ^7^ Theoretische Chemie TUD Dresden University of Technology Dresden Germany; ^8^ Center for Advanced Systems Understanding (CASUS) Helmholtz‐Zentrum Dresden‐Rossendorf Görlitz Germany; ^9^ Department of Chemistry and IBS for nanomedicine Yonsei University Seoul Republic of Korea; ^10^ Climate‐Neutral and Resource‐Efficient Construction (CARE) TUD Dresden University of Technology and RWTH Aachen Germany; ^11^ Centre for Tactile Internet with Human‐in‐the‐Loop (CeTI) TUD Dresden University of Technology Dresden Germany

**Keywords:** 2D conjugated metal–organic framework, anisotropic charge transport, chemiresistive sensing, ligand‐surfactant interaction, on‐water surface synthesis

## Abstract

Two‐dimensional conjugated metal–organic frameworks (2D *c*‐MOFs) offer an appealing platform for electronic devices, particularly chemiresistive sensors, owing to their unique combination of electrical conductivity and intrinsic porosity. However, their pronounced structural and transport anisotropies render device performance highly sensitive to layer orientation, underscoring the need for synthetic strategies that enable the controlled synthesis of well‐aligned 2D *c*‐MOF films. Here, we introduce a surfactant monolayer‐assisted on‑water synthesis that programs the layer orientation of conductive Ni‑HHTP (HHTP = 2,3,6,7,10,11‐hexahydroxytriphenylene) films (face‑on vs. edge‑on) over cm^2^‑scale areas by tuning ligand‐surfactant monolayer electrostatic vs. hydrogen‑bonding interactions. Imaging and scattering techniques unambiguously confirm preferential face‐on and edge‐on layer orientations, while electrical transport and optical pump‐THz probe spectroscopy reveal markedly enhanced intralayer charge transport in face‐on films, motivating their integration into chemiresistive sensing. Chemiresistive NH_3_ sensors based on face‑on Ni‑HHTP films achieve a response of 269.8% at 50 ppm and an ultralow detection limit of 8.45 ppb at room temperature, surpassing edge‑on films and previously reported 2D *c*‑MOF sensors. These results establish surfactant‐programmed on‐water synthesis as a new route to macroscopic orientation control in 2D *c*‐MOFs, enabling deliberate exploitation of their anisotropic charge transport in high‐performance sensing and electronic devices.

## Graphical abstract

Layer orientation dictates charge transport in 2D conjugated metal–organic frameworks, yet controllable methods remain scarce. We report surfactant monolayer‐assisted on‐water synthesis of cm^2^‐scale Ni‐HHTP films with programmable face‐on and edge‐on orientations. Tuning surfactant headgroups from quaternary ammonium to primary amines switches interfacial interactions, directing HHTP preorganization and orientation. Face‐on films show higher conductivity, mobility, and enhanced chemiresistive sensing performance.

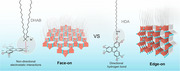

## 1 Introduction

Two‐dimensional conjugated metal–organic frameworks (2D *c*‐MOFs) represent a unique class of synthetic organic 2D crystals, in which *π*‐conjugated ligands and transition‐metal nodes can be programmably designed and interconnected to form layered lattice structures [1, 2, 3, 4, 5, 6]. By combining in‐plane *π*‐extended conjugation and out‐of‐plane van der Waals interactions, these materials uniquely integrate the intrinsic porosity of conventional MOFs with efficient charge transport [7, 8, 9, 10]. As a result, 2D *c*‐MOFs have emerged as promising functional materials for electronic applications, particularly in chemiresistive sensing, where high carrier mobility and accessible active sites are crucial [11, 12, 13, 14, 15, 16, 17, 18, 19]. However, 2D *c*‐MOFs are typically synthesized as bulk powders or polycrystalline films with randomly oriented domains, a structural limitation that significantly compromises device performance [4, 5, 20, 21, 22]. This challenge arises from the inherent electrical anisotropy of the 2D *c*‐MOF architecture, in which a fundamental imbalance exists between intralayer *π*‐conjugation and interlayer electronic coupling [23, 24, 25]. Therefore, the crystallographic orientation of the layers directly governs both the dominant charge‐transport pathways and the accessibility of active sites, highlighting the critical importance of layer‐orientation control.

Over the past decade, on‐surface synthesis (e.g., vapor/solid [26, 27, 28, 29], liquid/solid [30, 31, 32, 33, 34, 35]) and interfacial synthesis at the liquid/liquid interface or air/water interface (also known as on‐water surface synthesis) [5, 36, 37, 38], have been widely employed to synthesize high‐quality 2D *c*‐MOF films. By confining coordination reactions to 2D interfaces, these methods enable the synthesis of uniform, large‐area 2D *c*‐MOFs as monolayer, multilayer, or thick films. However, due to the absence of sufficient driving forces, these approaches typically lack control over ligand diffusion and preorganization, resulting in rapid, uncontrolled nucleation/crystallization kinetics [39]. As a result, the obtained films are typically polycrystalline, with limited domain sizes and, critically, poorly controlled layer orientations, often comprising a mixture of face‐on and edge‐on oriented grains [4, 22, 40]. Although several studies have reported liquid/solid interfacial approaches to 2D *c*‐MOF films with tunable layer orientation—achieved by modifying substrate surface chemistry, or changing the types of solvents/reaction temperature [30, 33, 41]—these systems suffer from limitations in film transferability, hindering their structural characterization and device integration. A promising avenue to overcome these synthetic challenges is the surfactant‐monolayer‐assisted interfacial synthesis (SMAIS) method [42, 43, 44, 45, 46, 47]. Originally developed for the synthesis of highly crystalline 2D Polymers, SMAIS leverages a surfactant monolayer (e.g., sodium oleyl sulfate) on the water surface to guide monomer preorganization via weak interactions, thereby directing 2D polymerization toward large‐area, single‐crystalline films with controlled layer orientation. Extending SMAIS to 2D *c*‐MOFs would offer a compelling opportunity to address the challenge of layer‐orientation control and to unlock their full potential in electronic devices.

Herein, we demonstrate the on‐water surface synthesis of Ni‐HHTP (HHTP = 2,3,6,7,10,11‐hexahydroxytriphenylene) films with controllable face‐on and edge‐on orientations via the SMAIS method. By selecting surfactants with quaternary ammonium or primary amine head groups, the ligand‐surfactant interactions can be tuned from predominantly electrostatic force to directional hydrogen bonding, thereby guiding the preorganization of HHTP into horizontal or vertical assemblies that dictate the crystallographic orientation of the resulting 2D *c*‐MOF films upon addition of Ni salt. The resulting Ni‐HHTP films are layer‐oriented over cm^2^‐scale areas, with thicknesses of ∼70 nm. Their crystal structure and preferential orientation were clearly resolved across both local and macroscopic scales by high‐resolution transmission electron microscopy (HRTEM) and grazing‐incidence wide‐angle x‐ray scattering (GIWAXS), respectively. Electrical and high‐frequency photoconductivity measurements reveal more favorable charge transport in face‐on Ni‐HHTP (i.e., Ni‐HHTP_DHAB, DHAB = dodecyl hexadecyl ammonium bromide) than in its edge‐on counterpart. Leveraging its efficient intralayer charge transport and vertically accessible active sites, Ni‐HHTP_DHAB was further integrated into chemiresistive gas sensors, demonstrating an exceptional NH_3_ sensing response of 269.8% at 50 ppm and an ultralow limit of detection (LOD) of 8.45 ppb at room temperature, surpassing edge‐on films and previously reported 2D *c*‐MOFs.

## 2 Discussion

### 2.1 Design and Controlled Synthesis of Oriented Ni‐HHTP Films

To achieve control over the layer orientation of 2D *c*‐MOFs, it is essential to introduce a driving force that preorganizes ligands into well‐defined horizontal or vertical assemblies prior to coordination polymerization. In this regard, the SMAIS method provides a compelling strategy, as it exploits a stable surfactant monolayer to adsorb and preorganize monomers through non‐covalent interactions, such as electrostatic forces and hydrogen bonding, between surfactant headgroups and monomers [42]. These interactions not only enrich the local monomer concentration on the water surface but also impose a predefined orientational bias that guides the subsequent growth of ordered 2D networks. In particular, non‐directional electrostatic interactions [48] between surfactant headgroups and monomers promote lateral accumulation and confinement of monomers, favoring extended, horizontal assemblies, whereas directional hydrogen‐bonding imposes geometric constraints that could bias the monomers toward vertical alignment [49]. By independently tuning these two interactions, rational control over the crystallographic orientation of 2D *c*‐MOFs—from face‐on to edge‐on configurations—can be achieved.

**FIGURE 1:**
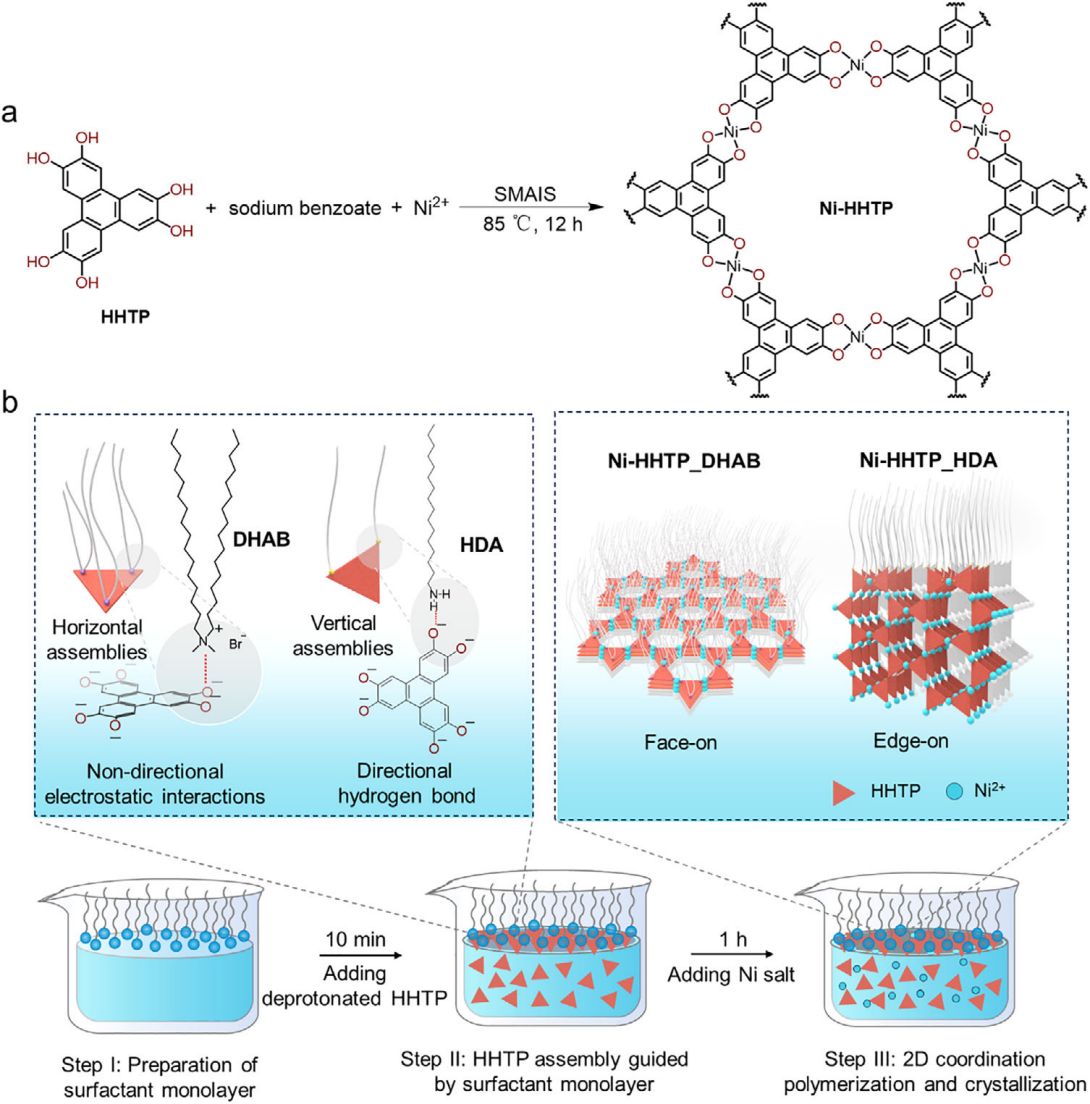
Schematic illustration of on‐water surface synthesis of Ni‐HHTP film with controllable layer orientation. (a) Schematic of the coordination reaction for Ni‐HHTP formation. (b) Preparation of face‐on and edge‐on oriented Ni‐HHTP thin films via the SMAIS method using different surfactants.

In this work, we followed a typical three‐step SMAIS procedure [42] (Figure 1b): (Step I) formation of a surfactant monolayer on the water surface; (Step II) introduction of the deprotonated ligand into the subphase; and (Step III) injection of a metal salt to initiate coordination polymerization. DHAB featuring positively charged quaternary ammonium headgroups, and hexadecylamine (HDA), with a neutral ─NH_2_ headgroup, were selected as surfactants to preorganize HHTP assemblies on the water surface via electrostatic interactions and hydrogen bonding. During the synthesis, a surfactant monolayer was first prepared on the water surface (pH = 8) by spreading a chloroform solution of DHAB or HDA surfactant (Figure S1). Time‐dependent surface pressure measurements confirmed that the surfactant layer remained stable on the water surface (pH = 8) for over 2 h, whereas it exhibited reduced stability on neutral water surface (pH = 7) (Figures S2 and S3a,b). After equilibration for 10 mins, 5 mL of an aqueous HHTP solution (6.2 × 10^−4^ m), containing sodium benzoate (3.1 × 10^−3^ m) as a deprotonating agent and modulator, was slowly added to the 40 mL water subphase. Note that the deprotonation of the HHTP ligand not only promotes electrostatic interactions with DHAB but also enforces the hydrogen bonding with HDA [50, 51]. Notably, a slight increase in surface pressure (from 41 to 44 mN/m for DHAB, from 24 to 38 mN/m for HDA) was observed after the addition of HHTP, indicating adsorption of ligand under surfactant monolayer (Figure S3c,d). After adding HHTP for 1 h, a thin film formed beneath the DHAB or HDA surfactant layer (Figure S4). Selected‐area electron diffraction (SAED) reveals a weak diffraction ring at 0.78 nm^−1^, corresponding to a *d*‐spacing of ∼1.3 nm, indicative of a preferentially horizontal (i.e., face‐on) assembly of HHTP ligands (Figure S5). In contrast, under otherwise identical conditions, HHTP assembled beneath the HDA monolayer into a film whose SAED pattern exhibits diffraction spots at 2.7 nm^−1^, consistent with a characteristic *π*–*π* stacking distance of ∼3.6 Å and suggesting a vertical (i.e., edge‐on) orientation (Figure S6). Density functional theory calculations in vacuum suggest that DHAB and HDA interact at faces or edges with neutral HHTP, respectively (Figure S7). However, unlike deprotonated HHTP, neutral HHTP exhibits intrinsically poor aqueous solubility and weak ligand‐surfactant interaction, limiting its diffusion and adsorption on the water surface. Therefore, deprotonated HHTP dominates the assembly and subsequent coordination polymerization on the water surface.

**FIGURE 2:**
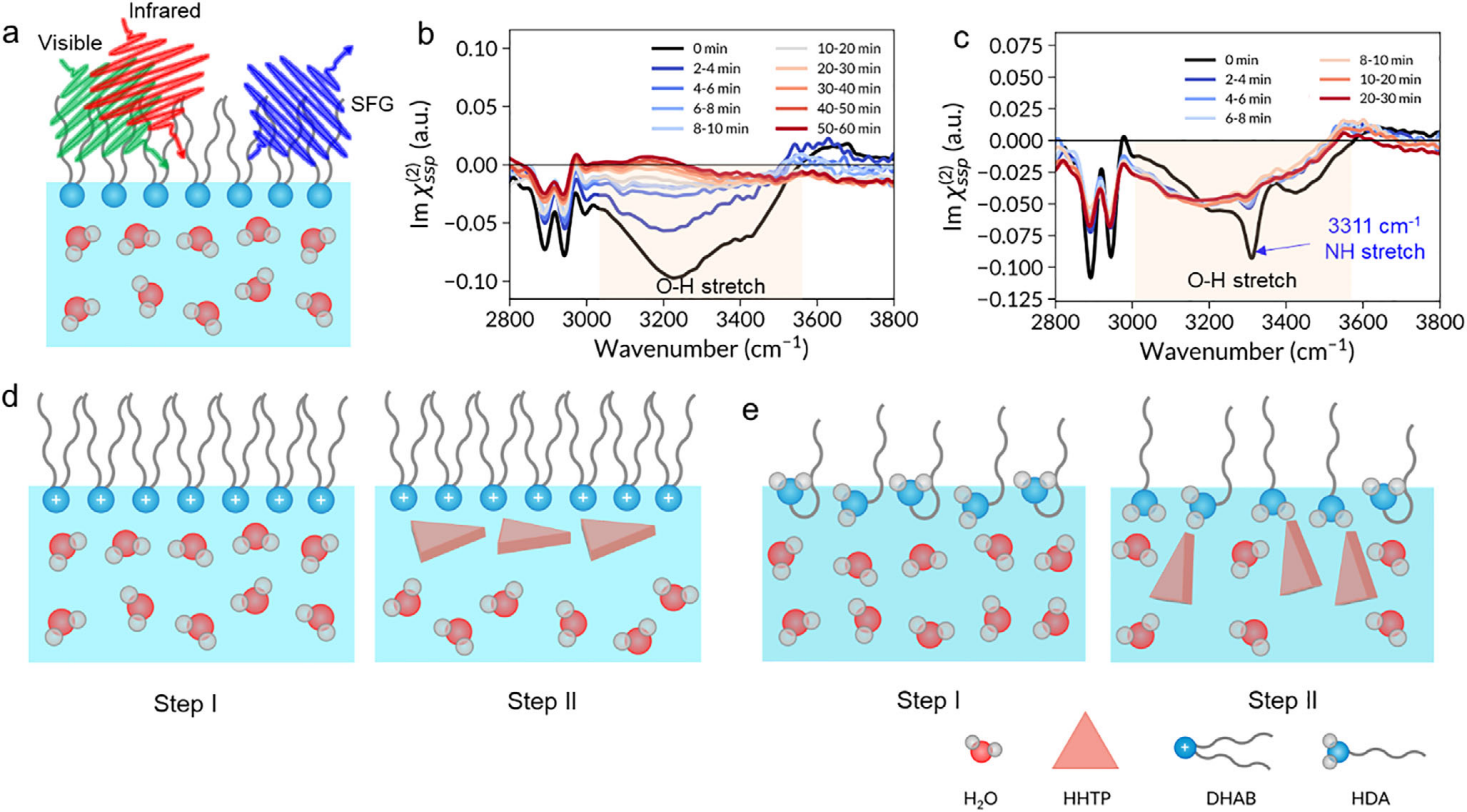
Time‐dependent surface‐specific vibrational SFG spectroscopy tracks the HHTP assembly behavior on the water surface using different surfactants. (a) The schematic illustration of the SFG measurement. (b) Imaginary χ^(2)^ spectra on the water surface for DHAB and HHTP assembly (face‐on pathway). The initially strong negative hydrogen‐bonded OH band (highlighted by light orange color) progressively weakens and reverses sign, indicating substantial restructuring of the interfacial field and water orientation upon formation of a laterally extended charged ligand layer. (c) Imaginary χ^(2)^ spectra on water interface for HDA and HHTP assembly (edge‐on pathway). The NH stretch at 3311 cm^−1^ disappears within minutes, and a redshifted shoulder develops in the OH region, consistent with headgroup reorientation and localized water‐ligand hydrogen bonding, without global inversion of the interfacial water network. (d) Schematic illustration of the DHAB‐driven assembly: electrostatic attraction promotes horizontal HHTP adsorption, strong screening of the surfactant field, and large‐scale reorganization of interfacial water. (e) Schematic illustration of the HDA‐driven assembly: directional hydrogen bonding promotes vertical ligand binding, mixed headgroup orientation, and localized modification of the hydrogen bond network.

To gain molecular‐level understanding of surfactant‐ligand assembly behavior, we monitored the time evolution of the surfactant monolayer on the water surface during ligand adsorption using heterodyne‐detected vibrational sum frequency generation (SFG) spectroscopy (Figure 2a) [52]. SFG spectroscopy selectively shows the net orientational order of interfacial molecules [53], so changes in the OH and NH stretching regions directly reflect how the surfactant headgroups and the adjacent water hydrogen bond network reorganize as the respective ligand accumulates beneath the surfactant monolayer [54, 55, 56].

We first measured the complex second‐order susceptibility on the water surface in the presence of the DHAB monolayer before HHTP injection, which was defined as 0 min in Figure 2b. The spectrum shows a strong negative hydrogen‐bonded OH band spanning ∼3000–3600 cm^−1^, consistent with a highly oriented interfacial water layer. This strong response is attributed to a positively charged quaternary ammonium monolayer, which generates an electric double layer with a strong interfacial electric field, thereby aligning the surrounding water molecules (Figure 2d). After HHTP injection, within ∼10 min, the hydrogen‐bonded OH region changes markedly, indicating strong disruption of the original water–DHAB interactions as HHTP displaces and reorganizes interfacial water. This supports the rapid adsorption of deprotonated HHTP at the interface via strong electrostatic attraction to the DHAB headgroups, annulling the double layer. At longer times, the OH response approaches a weakly positive Im χ^(2)^ in the hydrogen‐bonded region, indicating an inversion of the net water orientation relative to the initial state, suggesting that the effective interfacial field experienced by water has been strongly reduced. This behavior arises from partial displacement of interfacial water by an adsorbed ligand layer, screening of the DHAB charge by deprotonated HHTP, or the emergence of new hydrogen bonding motifs between water and the ligand layer (Figure 2d). Apparent changes in the CH region should be interpreted with care, as they can also arise from spectral interference with the broad OH continuum.

In contrast, the neutral −NH_2_ headgroup in HDA yields a qualitatively different initial interfacial spectrum. Before HHTP injection (0 min, Figure 2c), a sharp negative NH stretching band at 3311 cm^−1^ is observed, reflecting a net orientation of NH_2_ groups in HDA toward the air side (Figure 2e) [57]. The OH region is also negative but weaker than that of DHAB, reflecting the absence of a strong fixed interfacial charge. Upon HHTP injection, the NH feature disappears within ∼10 min, indicating reorganization of the −NH_2_ headgroup in HDA upon directional hydrogen bonding with the ligand. The hydrogen bonding is apparent from the emergence of a redshifted shoulder in the hydrogen‐bonded OH region. Notably, the overall OH amplitude remains relatively stable, implying that the water surfactant network is not disrupted as extensively as in the DHAB case, but is instead locally modified by water‐HHTP interactions. These observations provide direct spectroscopic evidence that the orientation of HHTP assembly on the water surface can be programmed by ligand‐surfactant interaction.

These SFG measurements directly distinguish electrostatic from hydrogen‐bonding interactions between the surfactant headgroups and HHTP. In the DHAB case (positively charged), the pronounced evolution of the hydrogen‐bonded OH response from a strong negative to a weakly positive Im χ^(2)^ signal demonstrates the gradual disappearance of the interfacial electric double layer upon addition of deprotonated HHTP, consistent with efficient screening of the positively charged quaternary ammonium monolayer by electrostatically bound HHTP ligands and concomitant displacement of interfacial water. In contrast, for charge‐neutral HDA, no electric double layer is present in the OH region, as would be expected for a DHAB‐like interaction mechanism. Instead, the OH amplitude remains largely unchanged while a redshifted shoulder emerges during HHTP adhesion, consistent with a local perturbation of the interfacial water structure rather than a global inversion. The most striking difference lies in the NH stretching band: its rapid disappearance upon HHTP injection is readily explained by the formation of strong NH···O hydrogen bonds between the amine headgroups and HHTP, which spectrally merges the NH stretch into the broader OH continuum. Together, the loss of the water double‐layer signature for HDA, the persistence of a relatively weak OH background, and the vanishing NH band provide direct spectroscopic evidence that DHAB engages HHTP predominantly via electrostatic attraction, whereas HDA interacts through localized hydrogen bonding, thereby establishing the mechanistic basis for the distinct face‐on and edge‐on assembly pathways.

Beyond these qualitative distinctions, the two systems also differ in both the spatial extent and kinetics of the interfacial transition. In the DHAB case, the initially strong OH response and its subsequent inversion indicate that the perturbation involves the formation and restructuring of an electrical double layer that extends several nanometers into the aqueous phase and evolves over a ∼10 min timescale. This reflects a collective, long‐range reorganization of interfacial water. In contrast, the HDA system exhibits a comparatively weak and nearly unchanged OH response, indicating that the perturbation is confined to the immediate interfacial region, limited to the first two to three molecular layers. The rapid disappearance of the NH feature suggests that hydrogen‐bond formation at the headgroup occurs on a faster timescale than the electrostatically driven restructuring in DHAB, but remains localized and does not propagate into the bulk‐like water network. Taken together, the DHAB system exhibits a spatially extended, field‐mediated transition with slower, collective kinetics, whereas the HDA system undergoes a faster, but spatially confined reorganization governed by short‐range hydrogen bonding.

### 2.2 Structural Characterization of Ni‐HHTP

**FIGURE 3:**
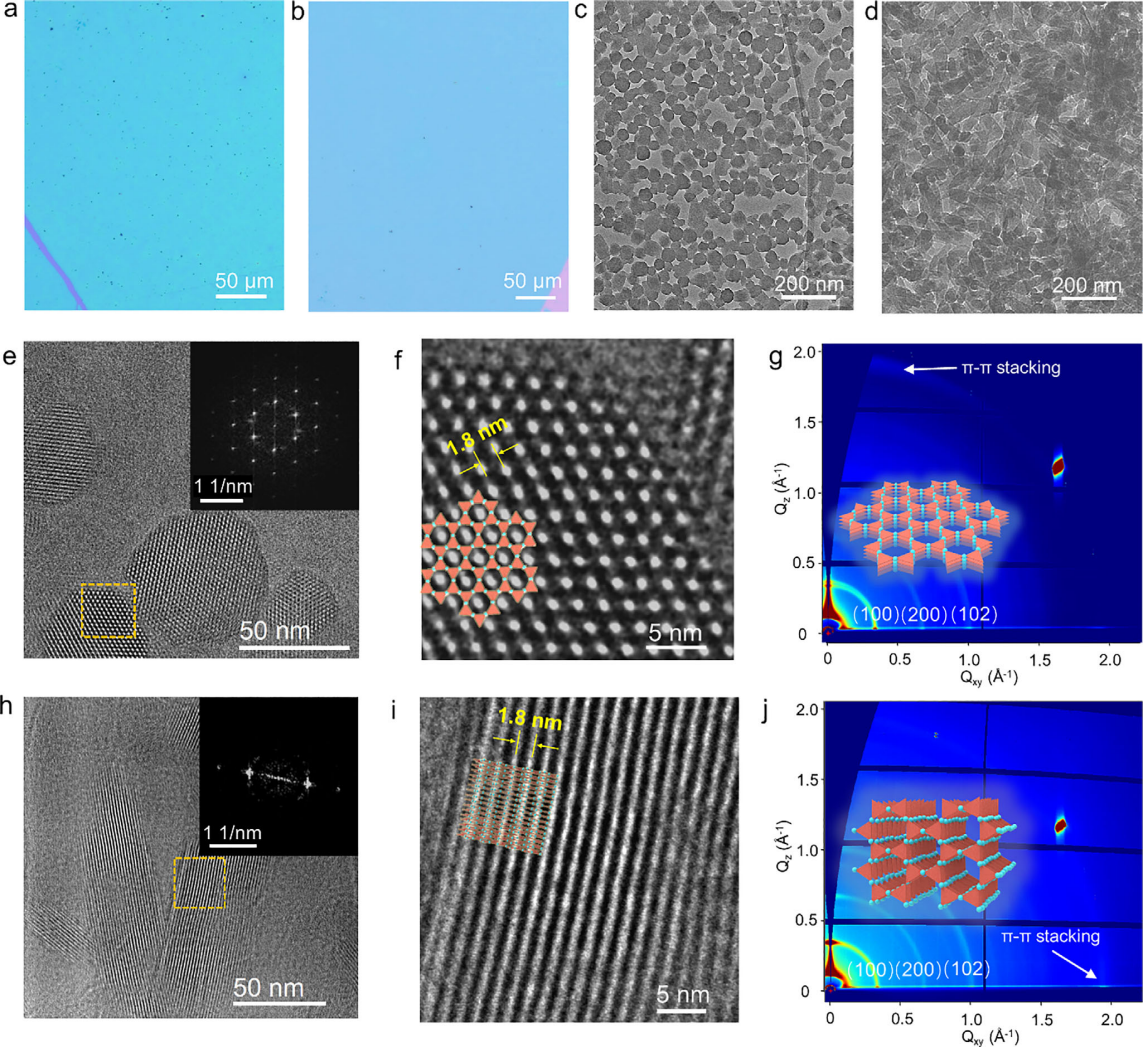
Morphological and structural characterization of Ni‐HHTP. (a,b) OM images of (a) Ni‐HHTP_DHAB and (b) Ni‐HHTP_HDA. (c,d) TEM images of (c) Ni‐HHTP_DHAB and (d) Ni‐HHTP_HDA. (e) HRTEM image of Ni‐HHTP_DHAB. Inset: fast Fourier transform (FFT) pattern from the yellow dashed region. (f) Enlarged HRTEM image of the yellow dashed region in (e), with the structural model overlaid. (g) GIWAXS diffraction pattern of Ni‐HHTP_DHAB. (h) HRTEM image of Ni‐HHTP_HDA. Inset: fast Fourier transform (FFT) pattern from the yellow dashed region. (i) Enlarged HRTEM image of the yellow dashed region in (h), with the structural model overlaid. (j) GIWAXS diffraction pattern of Ni‐HHTP_HDA.

After the preorganization of HHTP, 5 mL of aqueous Ni(OAc)_2_·4H_2_O (1.03 × 10^−3^ M) solution was injected into the subphase to initiate the coordination polymerization. The reaction was kept at 85°C under ambient conditions for 12 h, yielding homogeneous, cm^2^‐scale Ni‐HHTP films (denoted as Ni‐HHTP_DHAB and Ni‐HHTP_HDA when using DHAB and HDA as surfactant) on the water surface, as confirmed by optical microscopy (OM, Figure 3a,b; Figures S8 and S9) and scanning electron microscopy (SEM, Figure S10). Atomic force microscopy (AFM) measurements show that both films possess thicknesses of about 70–80 nm and are composed of densely packed crystalline domains (Figure S11). Attenuated total reflection Fourier transform infrared (ATR‐FTIR) spectroscopy reveals the disappearance of the ‐OH modes (∼3200 cm^−1^) from HHTP (Figure S12), confirming successful Ni and ligand coordination. SEM‐energy‐dispersive X‐ray spectroscopy mapping (SEM‐EDX) reveals a homogeneous distribution of Ni, O, and C elements across the film, indicating compositional homogeneity and consistent coordination throughout the sample (Figures S13 and S14).

To elucidate the crystal structure and layer orientation of the synthesized Ni‐HHTP films, we performed TEM and GIWAXS measurements. As shown in Figure 3c; Figure S15, the TEM image shows that the Ni‐HHTP_DHAB film is composed of flake‐like crystals with an average lateral size of ∼80 nm. HRTEM images reveal a well‐defined in‐plane honeycomb porous lattice with a *d*‐spacing of ∼1.8 nm (Figure 3e), in good agreement with previous reports (Figure 3f) [58]. The corresponding fast Fourier transform (FFT) pattern displays clear sixfold Bragg reflection spots from the in‐plane honeycomb structure, suggesting a preferential face‐on orientation (Figure 3e). This orientation assignment is further corroborated by GIWAXS measurements at the macroscopic scale. As shown in Figure 3g, the out‐of‐plane scattering profile exhibits a stronger reflection for the *π*–*π* stacking signal (*Q* = ∼1.95 Å^−^^1^) in the out‐of‐plane direction than in the in‐plane direction, indicative of a preferential face‐on configuration, i.e., layered stacking normal to the substrate. In addition, three Bragg reflections are observed at *Q* = 0.33, 0.66, and 1.00 Å^−1^, corresponding to the (100), (200), and (102) lattice planes, respectively, which are in good agreement with the reported crystal structure of Ni‐HHTP obtained via solvothermal synthesis [58]. In contrast, the Ni‐HHTP_HDA film exhibits a markedly different morphology and orientation, consisting of rod‐like crystallites with lengths of 100–200 nm (Figure 3d; Figure S16). HRTEM images reveal highly ordered lattice fringes with a *d*‐spacing of ∼1.8 nm, which is consistent with the in‐plane (100) periodicity of Ni‐HHTP (Figure 3h,i). The corresponding FFT patterns display linear Bragg reflection spots, indicating a preferential edge‐on orientation at the nanoscale (Figure 3h). Consistently, the GIWAXS pattern displays a more pronounced *π*–*π* stacking reflection at *Q* = ∼1.93 Å^−1^ in the in‐plane direction (Figure 3j), confirming that the layered stacking is predominantly parallel to the substrate, that is, an edge‐on configuration. Furthermore, there are three strong Bragg reflections at *Q* = 0.34, 0.68, and 1.01 Å^−1^, indicating almost the same crystal structure as in Ni‐HHTP_DHAB [58]. These results demonstrate that the Ni‐HHTP_DHAB and Ni‐HHTP_HDA films adopt predominantly face‐on and edge‐on orientations, respectively, over macroscopic areas, highlighting the key role of HHTP‐surfactant interactions in programmable orientation control of 2D *c*‐MOF films. We note that DHAB exhibits lower effective head‐group densities compared with single‐chain surfactants or shorter‐chain (e.g., cetyltrimethylammonium bromide and didodecyldimethylammonium bromide), which suppresses nucleation density and promotes the formation of larger crystalline domains (Figures S17 and S18).

### 2.3 Anisotropic Charge Transport Properties in Ni‐HHTP

**FIGURE 4:**
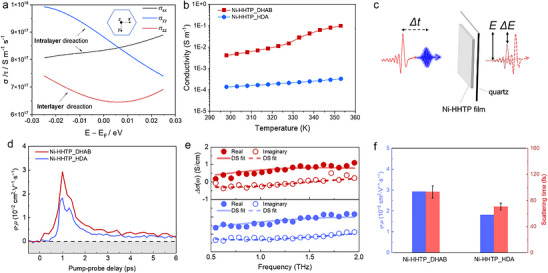
Anisotropic charge transport in Ni‐HHTP. (a) Calculated electrical conductivity along different directions within the constant‐relaxation‐time approximation of the Boltzmann transport equation using the BoltzTraP2 code. (b) Experimental temperature‐dependent conductivity measurements for Ni‐HHTP_DHAB and Ni‐HHTP_HDA films. (c) Schematic illustration of probing microscopic charge transport properties in Ni‐HHTP films using OPTP spectroscopy. (d) THz photoconductivity dynamics for Ni‐HHTP_DHAB and Ni‐HHTP_HDA under 3.10 eV photoexcitation at a pump fluence of ∼0.8 mJ cm^−2^. (e) Frequency‐resolved complex photoconductivity spectra of Ni‐HHTP_DHAB (top) and Ni‐HHTP_HDA (bottom), measured at 0.5 ps after the peak photoconductivity. (f) Comparison of the effective charge carrier mobility (left axis) and scattering time (right axis) for Ni‐HHTP_DHAB and Ni‐HHTP_HDA.

To elucidate the intrinsic anisotropy of charge transport in Ni‐HHTP, we first evaluated its direction‐dependent electrical conductivity using Boltzmann transport theory within the constant relaxation time approximation, as implemented in the BoltzTraP2 code. The calculation indicates that the intralayer conductivities (*σ*_xx_ and *σ*_yy_) exceed the interlayer conductivity (*σ*_zz_), establishing Ni‐HHTP as an inherently anisotropic charge‐transport system (Figure 4a; Figure S19). Guided by this theoretical insight, we measured the optical bandgap and temperature‐dependent electrical conductivity of both Ni‐HHTP_DHAB and Ni‐HHTP_HDA films. UV–vis absorption spectra (Figure S20) indicate an optical bandgap of 1.63 eV for Ni‐HHTP_DHAB, slightly smaller than the 1.70 eV measured for Ni‐HHTP_HDA, suggesting that the face‐on oriented domains in Ni‐HHTP_DHAB favor enhanced conjugation compared to the edge‐on configuration. Further experimental variable‐temperature conductivity measurements using a two‐probe method reveal a nonlinear increase in electrical conductivity for both films from 298 to 353 K (Figure 4b), characteristic of semiconducting behavior. Notably, at room temperature, the Ni‐HHTP_DHAB film exhibits a conductivity of 4.08 × 10^−3^ S m^−1^, over one order of magnitude higher than that of the Ni‐HHTP_HDA film (1.39 × 10^−4^ S m^−1^). This pronounced difference can be attributed to the preferential face‐on orientation in Ni‐HHTP_DHAB, which facilitates more efficient intralayer charge transport through continuous *π*‐d pathways, whereas the edge‐on orientation in Ni‐HHTP_HDA imposes slower interlayer hopping pathways that limit conductivity.

To examine the intrinsic charge transport properties of Ni‐HHTP_DHAB and Ni‐HHTP_HDA films, we further performed high‐frequency photoconductivity measurements using optical pump‐THz probe (OPTP) spectroscopy (Figure 4c). An ultrashort pump pulse (3.1 eV, ∼100 fs) photoinjects charge carriers by promoting interband transitions. A time‐delayed, linearly polarized single‐cycle THz electric field (∼1 ps duration) subsequently drives the photoinduced carriers and probes their local charge transport properties [59]. For Ni‐HHTP_DHAB, which adopts a predominantly face‐on orientation, the THz probe primarily senses intralayer charge transport. By contrast, in edge‐on‐oriented Ni‐HHTP_HDA, the THz probe captures a mixed response that incorporates both interlayer and intralayer charge‐transport components along the THz polarization direction. Figure 4d shows the THz photoconductivity dynamics plotted in units of effective mobility (*ϕ*·*μ*), reflecting the product of the free carrier generation quantum yield (*ϕ*) and the charge mobility (*μ*). Upon photoexcitation, both Ni‐HHTP_DHAB and Ni‐HHTP_HDA show a rapid rise within ∼1 ps, indicating the formation of mobile carriers, followed by a few‐picosecond decay that likely arises from carrier trapping and/or electron‐hole recombination [60]. Ni‐HHTP_DHAB shows a larger effective mobility than Ni‐HHTP_HDA (2.9 ± 0.1 × 10^−2^ vs. 1.8 ± 0.1 × 10^−2^ cm^2^·V^−1^·s^−1^), indicating that the intralayer direction provides more efficient pathways for free carrier generation and/or charge migration. This observation is consistent with electrical conductivity measurements and highlights the anisotropic charge transport in Ni‐HHTP. To gain more quantitative insight, we measured the frequency‐dependent complex photoconductivity. As displayed in Figure 4e, both samples exhibit a suppressed positive real component and a negative imaginary component at low frequencies, hallmarks of spatially restricted carrier motion described by the Drude‐Smith (DS) model:

$$\Delta \sigma \left(\omega \right)=\frac{\omega_{p}^{2}\varepsilon_{0}\tau_{DS}}{1-i\omega \tau_{DS}}\left(1+\frac{c}{1-i\omega \tau_{DS}}\right)$$

where ω_*p*_ is the plasma frequency, ε_0_ is the vacuum permittivity, τ_*DS*_ is the DS scattering time, and *c* ranging from −1 to 0 is the backscattering parameter (*c* = −1 indicates fully preferential backscattering, while *c* = 0 corresponds to isotropic momentum randomization). Fitting the spectra yields τ_*DS*_ values of 93 ± 10 and 71 ± 5 fs, and *c* parameters of −0.9 for Ni‐HHTP_DHAB and Ni‐HHTP_HDA, respectively. The nonzero *c* values are consistent with the polycrystalline nature of the films. We find that the extracted effective mobility and τ_*DS*_ follow the same trend (Figure 4f), suggesting that reduced charge scattering is likely the primary origin of the more efficient charge transport along the intralayer direction. Together, these results unambiguously demonstrate that intralayer transport is intrinsically more favorable than interlayer transport in Ni‐HHTP, highlighting the critical role of crystallographic orientation in governing charge transport properties in 2D *c*‐MOFs.

Taken together, the DC conductivity, OPTP‐derived mobility, and optical bandgap paint a consistent picture of orientation‐controlled transport in Ni‐HHTP. At room temperature, the face‐on Ni‐HHTP_DHAB film exhibits more than an order‐of‐magnitude higher DC conductivity than the edge‐on Ni‐HHTP_HDA film, despite the THz‐range effective mobilities differing only by a factor of ∼1.6, indicating that macroscopic charge transport is additionally limited by a higher density of traps and/or less favorable percolation pathways in the edge‐on configuration. This interpretation is supported by the Drude–Smith analysis, where similar backscattering parameters but shorter scattering times in Ni‐HHTP_HDA point to stronger carrier localization and enhanced scattering at grain boundaries and defects. The slightly narrower optical bandgap of Ni‐HHTP_DHAB further suggests enhanced intralayer conjugation, which facilitates carrier delocalization within the *π*–d planes and lowers the energetic penalty for charge transport along the in‐plane direction. Thus, while OPTP probes the intrinsic, sub‐picosecond mobility of photogenerated carriers within individual domains, the DC measurements integrate over long‐range transport across intergrain barriers, and the systematic differences between the two films in both regimes consistently underscore that intralayer transport in the face‐on architecture is intrinsically more favorable than the predominantly interlayer‐dominated pathways in edge‐on films.

### 2.4 Chemiresistive Sensing Properties

**FIGURE 5:**
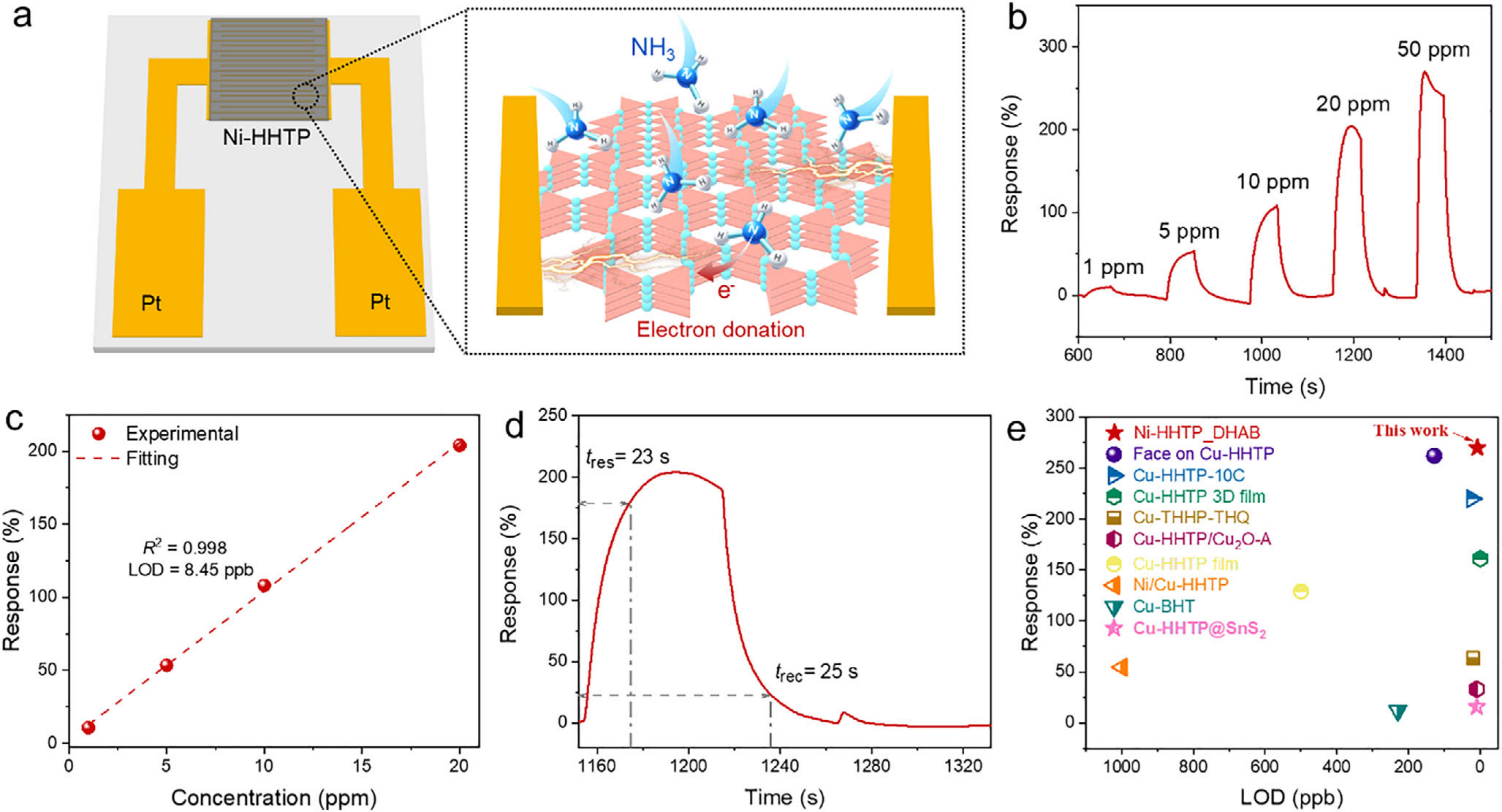
Room temperature NH_3_ sensing performances of the Ni‐HHTP_DHAB film. (a) Scheme of chemiresistive sensors based on Ni‐HHTP_DHAB film. (b) Real‐time sensing response curve of Ni‐HHTP_DHAB‐based gas sensors toward NH_3_ exposure from 1 to 50 ppm. (c) The linear relationship between response and NH_3_ concentration of Ni‐HHTP_DHAB samples. (d) Response and recovery times calculated for 20 ppm NH_3_ of Ni‐HHTP_DHAB. (e) Comparison of sensing performance metrics between our materials‐based gas sensors and previously reported materials‐based gas sensors.

In chemiresistive sensors, both charge‐transport efficiency and analyte accessibility jointly govern signal transduction: high conductivity and carrier mobility enable rapid, amplified resistance modulation, whereas exposed active sites promote effective analyte adsorption and charge transfer. Leveraging its efficient intralayer charge transport and the high accessibility of its active sites, the Ni‐HHTP_DHAB film was integrated into chemiresistive devices by depositing onto pre‐patterned Pt electrode chips (channel width of 4 µm) (Figure 5a), serving as a proof‐of‐concept demonstration. As shown in Figure 5b, the device exhibits pronounced, concentration‐dependent responses to NH_3_ over 1–50 ppm. In all cases, NH_3_ exposure induces an increase in resistance, consistent with a hole‐dominated (p‐type) sensing mechanism [61]. Remarkably, the Ni‐HHTP_DHAB‐based sensor shows a response increasing from 10.7% to 269.8% over 1–50 ppm, superior to the Ni‐HHTP_HDA (increase from 6.6% to 220.1%) (Figure S21), Ni‐HHTP in powder form (Figures S22 and S23), and most of the reported 2D *c*‐MOFs (Table S1).

At low NH_3_ concentrations, gas molecules preferentially adsorb at pore‐accessible active sites (i.e., Ni─O coordinated sites) (Figure S24), where even limited adsorption effectively modulates the carrier concentration. With increasing NH_3_ concentration, the vertically aligned channels in the face‐on configuration become progressively filled, enabling continuous response enhancement, whereas the edge‐on configuration offers fewer effective adsorption sites and thus saturates more rapidly. Consequently, the Ni‐HHTP_DHAB sensors achieve an ultralow LOD of 8.45 ppb (Figure 5c), significantly outperforming the Ni‐HHTP_HDA device (34.2 ppb, Figure S25). Moreover, under 20 ppm NH_3_, the Ni‐HHTP_DHAB device exhibits a fast response time (*t*_res_, the time required for the current to decrease to 90% of its saturation value [62]) of 23 s and a recovery time (*t*_rec_, the time needed for the current to increase to 10% of its original value) of 25 s (Figure 5d), faster than Ni‐HHTP_HDA (*t*_res_ = 35 s*, t*_re_ *_c_* = 28 s) (Figure S26). In addition, the Ni‐HHTP_DHAB device exhibits markedly improved operational stability during repeated gas sensing cycles compared to Ni‐HHTP_HDA, maintaining stable and reproducible resistance responses with negligible baseline drift. In contrast, Ni‐HHTP_HDA shows pronounced signal attenuation and progressive baseline shift upon cycling, indicating gradual performance degradation (Figures S27 and S28). As summarized in Figure 5e and Table S1, the Ni‐HHTP_DHAB sensor exhibits a high response (269.8% @50ppm) and an ultralow LOD of 8.45 ppb, representing one of the best performances reported [63]. These enhanced performance metrics arise from the face‐on orientation, which provides vertically accessible channels that facilitate efficient gas diffusion, delay saturation, and maximize the coupling between anisotropic charge transport and orientation‐dependent active‐site accessibility.

## 3 Conclusion

In summary, we demonstrated the novel on‐water surface synthesis of Ni‐HHTP with controllable layer orientation using the SMAIS method. By rationally selecting surfactants that modulate the dominant HHTP‐surfactant interactions from non‐directional electrostatic force to directional hydrogen bonding, the growth of Ni‐HHTP can be programmatically switched between face‐on and edge‐on configurations. Comprehensive structural characterization by HRTEM and GIWAXS unambiguously confirms this orientation control across both nanoscale and macroscale. Electrical and OPTP measurements reveal pronounced charge transport anisotropy, showing that face‐on oriented Ni‐HHTP_DHAB exhibits higher conductivity and enhanced carrier mobility compared to its edge‐on counterpart. When integrated into chemiresistive sensors, Ni‐HHTP_DHAB delivers an exceptional NH_3_ response of 269.8% at 50 ppm and an ultralow LOD of 8.45 ppb at room temperature, outperforming Ni‐HHTP_HDA and previously reported 2D *c*‐MOFs. This work opens up new opportunities for controlled synthesis of layer‐oriented 2D *c*‐MOFs, enabling the exploitation of their structural and transport anisotropies for high‐performance electronic devices.

## 4 Experimental Section

### 4.1 Synthetic Conditions of Ni‐HHTP_DHAB

10 µL DHAB (1 mg/mL in CHCl_3_) was spread onto the surface of 40 mL deionized water (pH = 8) in a beaker, then 5 mL aqueous solution of HHTP (6.2 × 10^−4^ M) containing sodium benzoate (3.1 × 10^−3^ M) was slightly added to the beaker. After 1 h, 5 mL of Ni(OAc)_2_·4H_2_O (1.03 × 10^−3^ m) solution was injected into the beaker. The beaker was kept at 85°C for 12 h.

### 4.2 Synthetic Conditions of Ni‐HHTP_HDA

10 µL HDA (1 mg/mL in CHCl_3_) were spread onto the surface of 40 mL deionized water (pH = 8) in a beaker, then 5 mL aqueous solution of HHTP (6.2 × 10^−4^ m) containing sodium benzoate (3.1 × 10^−3^ m) was slightly added to the beaker. After 1 h, 5 mL of Ni(OAc)_2_·4H_2_O (1.03 × 10^−3^ m) solution was injected into the beaker. The beaker was kept at 85°C for 12 h.

### 4.3 Film Transfer

After synthesis, the films were transferred onto various substrates (e.g., SiO_2_/Si, Si, quartz, and Cu grids) using a dipping method. In this process, the substrate was first immersed in the subphase beneath the film and then slowly lifted to bring it into contact with the film, thereby transferring the film onto the substrate surface. The transferred films were subsequently rinsed three times with water, DMF, CHCl_3_, and acetone, followed by vacuum drying at 80°C for 24 h.

### 4.4 HD‐SFG Spectroscopy

The respective systems were characterized using a heterodyne‑detected sum‑frequency generation (HD‑SFG) spectrometer. Detailed descriptions of the instrument can be found elsewhere [54, 64]. In brief, the setup was driven by a 1 kHz Spitfire Ace Ti: Sapphire regenerative amplifier (Spectra Physics) operating at 800 nm. The output beam was divided into two paths: one arm was routed through a grating–cylindrical mirror pulse‑shaping assembly to generate a narrowband visible pulse (∼15 cm^−1^), while the second arm was converted into a broadband mid‑infrared pulse via an optical parametric amplifier (Light Conversion TOPAS‑C, Spectra Physics) followed by difference‑frequency generation in an AgGaS_2_ crystal. Both beams were then spatially overlapped and focused onto a 20 µm y‑cut quartz plate to produce the local oscillator (LO). A 2‑mm SrTiO_3_ plate was inserted upstream of the sample to introduce a controlled phase shift. The LO, together with the visible and mid‑IR pulses, was directed onto the sample at an incident angle of 45°. Interference between the sample‑generated SFG signal and the LO produced an interferogram, which was dispersed by a spectrometer (Teledyne Princeton Instruments, HRS‑300) and detected using a liquid‑nitrogen‑cooled CCD (Teledyne Princeton Instruments, PyLoN). All measurements were performed under dry, purged conditions. The complex χ^(2)^ spectra were retrieved by Fourier transforming the interferograms and subsequently normalized to the response of a z‑cut quartz reference [53]. Experiments were conducted under N_2_ using the ssp polarization combination, where ssp corresponds to s‑polarized SFG, s‑polarized visible, and p‑polarized mid‑IR fields. During the measurement, the deprotonation agent was changed from sodium benzoate to NaOH to reduce the signal originating from sodium benzoate.

### 4.5 Gas Sensing Measurement

All sensing measurements were carried out in a sealed chamber with a volume of 100 cm^3^ at room temperature [65, 66]. The target gases were bought from Air Products (UK) and were pre‐diluted in dry nitrogen. The desired analyte concentrations were accurately controlled using a gas mixing and flow control system (MCQ Instruments, GB‐103), while the total gas flow rate was maintained at 500 sccm throughout the measurement. The target gas measuring process includes 1 min (60 s) of exposure and 2 min (120 s) of flushing with N_2_, respectively. Before testing, we use N_2_ to acquire a stable baseline for 10 min. For the testing process, the sensor was powered with a constant bias voltage of 3.0 V, and real‐time electrical resistance signals were recorded by using a Keithley 2450 source meter. Sensor response was defined as the relative resistance change: S (%)  =  Δ*R*/*R*0  ×  100  =  (*Rt*–*R*0)/*R*0  ×  100, where *R*_0_ is the initial resistance in nitrogen, and *R_t_* is the real‐time resistance exposed to the target gases. Response and recovery times were defined as the time to reach 90% of the total resistance change during gas exposure and flushing, respectively.

## Conflicts of Interest

The authors declare no conflicts of interest.

## Supporting information




**Supporting File**: adma73785‐sup‐0001‐SuppMat.pdf.

## Data Availability

The data that support the findings of this study are available from the corresponding author upon reasonable request.
