# Supplementary material for: On‐Water Surface Synthesis of 2D Conjugated Metal–Organic Framework Films With Controllable Layer Orientation Enabling High‐Performance Chemiresistive Sensing
Source: Adv Mater. 2026 Jun 23;38(41):e73785. doi: 10.1002/adma.73785 (PMC13393957; doi:10.1002/adma.73785)
Supplement: Supplementary file 1 — Supporting File: adma73785‐sup‐0001‐SuppMat.pdf. [file ADMA-38-e73785-s001.pdf]

Supporting Information

On-water Surface Synthesis of 2D Conjugated Metal-Organic Framework Films with Controllable Layer Orientation Enabling High-performance Chemiresistive Sensing

Materials and methods

All chemicals were purchased from commercial sources and were used without any further purification. Optical Microscopy (OM) was performed using an AxioScope A1, Zeiss. Scanning electron microscopy (SEM) was performed using a Gemini 500 microscope (Carl Zeiss, Germany) equipped with an energy-dispersive X-ray (EDX) detector. Transmission electron microscopy (TEM) images were acquired using a JEOL JEM-F200 microscope (JEOL Ltd., Japan) operated at 200 kV. Samples were deposited onto copper grids prior to TEM analysis. Fourier transforms infrared (FT-IR) spectra were collected using Tensor II (Bruker) with an attenuated total reflection (ATR) unit. Ultraviolet-Visible (UV-vis) spectra were recorded on an Agilent Cary 5000 UV-VIS-NIR. Atomic force microscopy (AFM) images were recorded using a Park Systems NX10 instrument. Surface pressure measurements were performed using a Langmuir-Blodgett trough (KSV NIMA, Finland) equipped with a platinum Wilhelmy plate. Grazing-incidence wide-angle X-ray scattering (GIWAXS) measurements were performed at beamline XRD1 at ELETTRA, Trieste, Italy. The energy of the beam was 12.4 keV and the beam had a diameter of 200 μm. Images were recorded using a Dectris Pilatus 2M area detector, which was placed 401 mm behind the sample. The sample-to-detector distance and beam center on the detector were verified using a lanthanum hexaboride standard. The incidence angle was 0.12° and the samples were exposed for 120 s to the beam while being rotated for 360°. The recorded data was corrected and analyzed using WxDiff.

Gas phase ligand-surfactant interaction calculations were performed using density-functional theory (DFT) employing the GGA-PBE exchange-correlation functional [1-PBE][1] in combination with the Grimme3 dispersion correction [2-Grimme][2] and a TZ2P basis set, considering all valence and core electrons implemented within the AMS code [3-AMS].[3] The surfactants HDA and DHAB were approximated by short-chain amine and ammonium molecules, and the geometries of the HHTP-surfactant complexes were optimized in the gas phase starting from initial face-on and edge-on orientations.

Electronic structure and conductivity calculations

DFT calculations were carried out using the Vienna ab-initio Simulation Package (VASP)4,5 version 5.4.1. The electronic wave-functions were expanded in a plane-wave basis set with a kinetic energy cutoff of 600 eV. The convergence criterion for geometry optimization was set such that the forces acting on the ions were smaller than 0.02 eV $Å^{-1}$. Electron-ion interactions were described using the projector augmented wave (PAW) method.[4] Generalized gradient

approximation (GGA) of the exchange-correlation energy in the form of Perdew-Burke-Ernzerhof (PBE) was applied.[5] We used the DFT+U approach to describe the localized d-orbitals of Ni ions. The effective Coulomb (U) and exchange (J) terms were set to 4 and 1 eV,[6] respectively. Such an approach was already successfully applied for similar systems. A Monkhorst-Pack Gamma-centered grid with 1×1×1 dimensions was used for K-point sampling of the Brillouin zone for geometry optimization and it was extended to 3×6×3 for the band structure calculations. The DFT-D3 method with Becke-Johnson damping function for the interlayer dispersion interactions was applied.[7] Geometry optimizations were performed with the cell vectors fixed to the experimental CIF values, and water solvent molecules were removed to reduce the computational cost.[8] In order to determine the full set of high-symmetry K-points in the Brillouin zone, a VASPKIT[9] code for pre- and post-processing of the VASP calculated data was used. The electrical conductivity (σ) was calculated at 300 K within the framework of the Boltzmann transport equation using the BoltzTraP2 code.[10] The k grid of the VASP calculations was interpolated onto a 5-times denser grid.

NH_3 adsorption calculations

All calculations were performed using Gaussian 16 (Rev.C.01) [1-Gaussian],[2] with PBE0 functional[5] and D3 version of Grimme's dispersion corrections with Becke-Johnson damping (D3BJ)[7] and Ahlrich's Def2-TZVPP basis set. Structures were adapted from those used previously for theoretical conductivity calculations. HHTP was passivated with hydroxyl (-OH) functional groups at two of the three Ni positions. Adsorption free energy ΔG_{ads} at the surface-accessible and pore-accessible active sites, and at the possible bare nickel defects arising from inhomogeneities in the HHTP monomer distribution between layers were calculated as,

$$\Delta G_{ads} = \Delta E_{ads} + \Delta G_{corrections},$$

where $G_{corrections}$ accounts for the correction from electronic energies to free energies, E_{ads} are the electronic energies and differences are calculated for each term as,

$$\Delta E_{ads} = E_{System-NH_3} - E_{NH_3} - E_{System}.$$

Optical pump-THz probe (OPTP)

The OPTP system is driven by a regenerative amplified, mode-locked Ti:sapphire laser producing ultrashort pulses with a central photon energy of 1.55 eV, a pulse duration of ~50 fs, and a repetition rate of 1 kHz. The output beam is divided by beam splitters into three optical paths for photoexcitation, THz generation, and THz detection. For optical excitation, the fundamental output is frequency-doubled to 3.10 eV using a β-barium borate (BBO) crystal.

Single-cycle THz pulses with a temporal width of ~1 ps are generated by optical rectification of the fundamental 1.55 eV pulses via a 1-mm-thick <110> ZnTe crystal. The emitted THz radiation is collected, collimated, and focused onto the sample using a pair of 90° off-axis parabolic mirrors. After transmission through the sample, a second pair of 90° off-axis parabolic mirrors collects and refocuses the THz pulses onto a second 1-mm-thick <110> ZnTe crystal for THz detection. The time-dependent THz electric field is mapped using a mechanical delay stage via free-space electro-optic sampling. The resulting differential signal is filtered and amplified with a lock-in amplifier for data acquisition. All measurements are performed in a dry N_2-purged environment to minimize THz absorption by atmospheric water vapor.

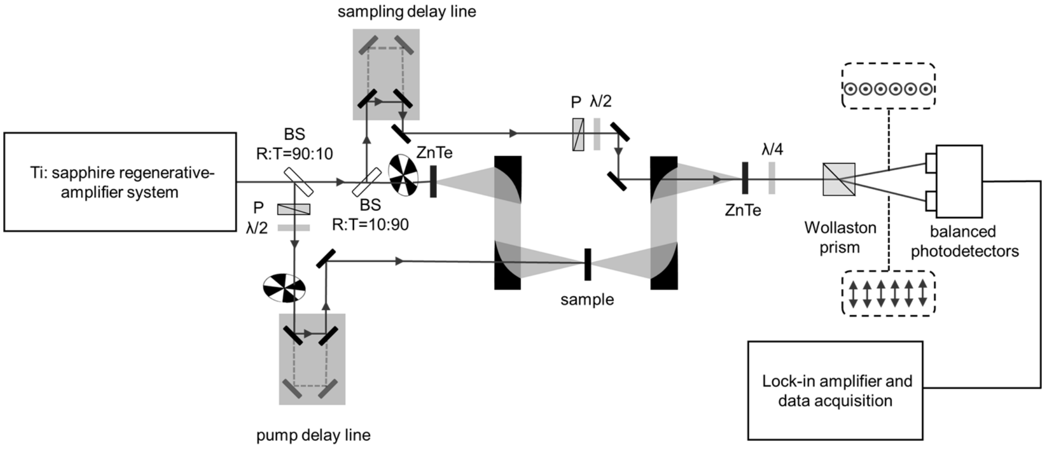

Scheme S1. Schematic of the OPTP setup. P, λ/2, λ/4, and BS denote a polarizer, half-wave plate, quarter-wave plate, and beam splitter, respectively.[11]

Fabrication of single-channel sensors:

Firstly, the silicon substrate was treated by bath sonication with acetone, isopropanol, and deionized water for 5 min, respectively. The spin coating method was used to deposit photoresist. After drying, it is exposed via UV lithography and developed for 5 min. After that, around 50 nm thickness Pt was deposited via the magnetron sputtering method. Lastly, the lift-off process was achieved with the acetone bath sonicating, and then we got the Interdigitated Electrodes (IDE) single-channel sensors. The width of the channel is 4 μm, gap of the channel is 3 μm. The Ni-HHTP films on the IDE sensor were fabricated by depositing the film on the pre-patterned substrate, and washing with $CHCl_3$, EtOH and acetone. Subsequently, the samples were dried in the air.

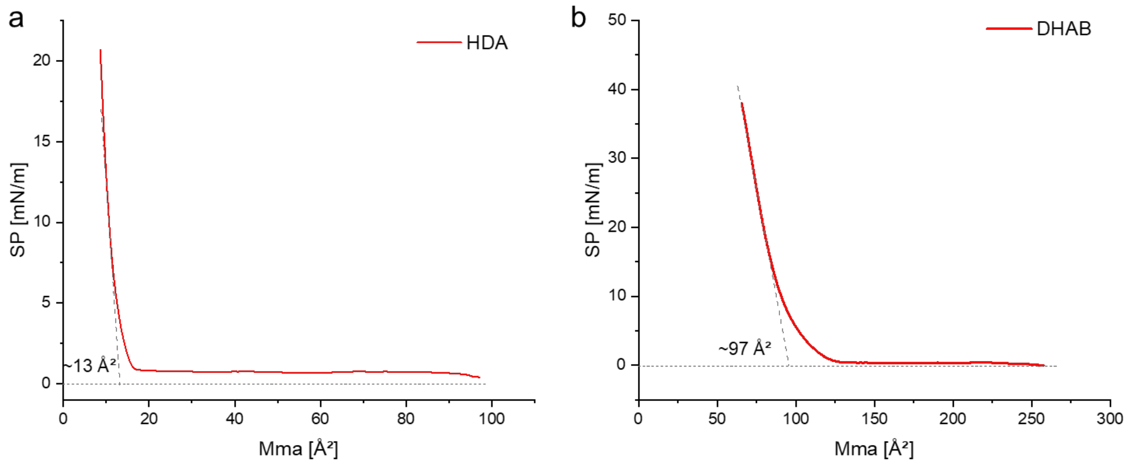

Figure S1. Surface pressure-mean molecular area isotherm of the surfactant monolayers on deionized water (pH = 8) and surfactant monolayers

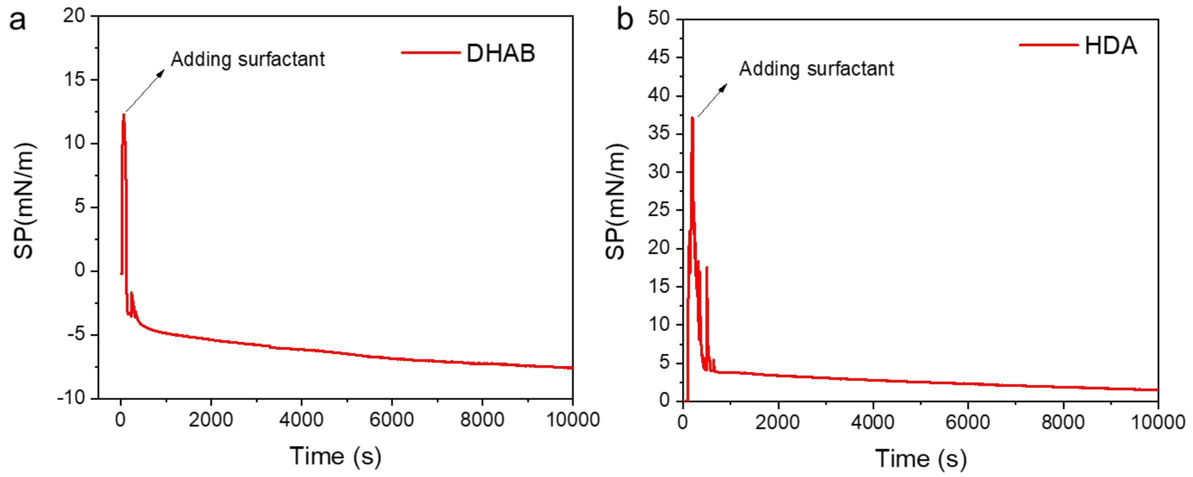

Figure S2. Isotherm curves of surface pressure versus time measured on the water surface (pH = 7) for (a) DHAB, (b) HDA. The surface pressures of both DHAB and HDA decrease dramatically over time, indicating their poor stability.

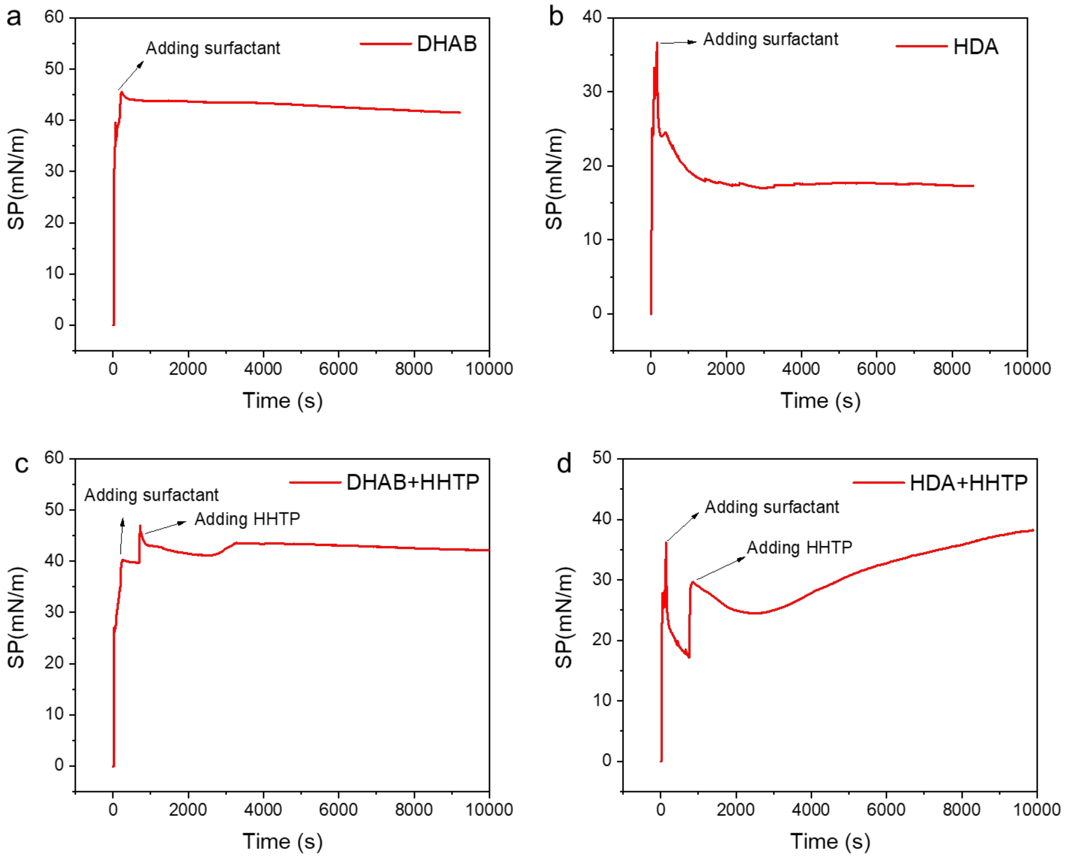

Figure S3. Isotherm curves of surface pressure versus time measured on the water surface (pH = 8) for (a) DHAB, (b) HDA, (c) DHAB+HHTP, and (d) HDA+HHTP. As shown in Figures S3a and S3b, DHAB and HDA are stable on the basic water surface. Upon introduction of deprotonated HHTP, the surface pressure increases progressively with time and subsequently reaches a steady state. This behavior suggests the formation of a more stable interfacial assembly, which can be attributed to interactions between the surfactant molecules and HHTP, resulting in a stabilized and preorganized film on the water surface.

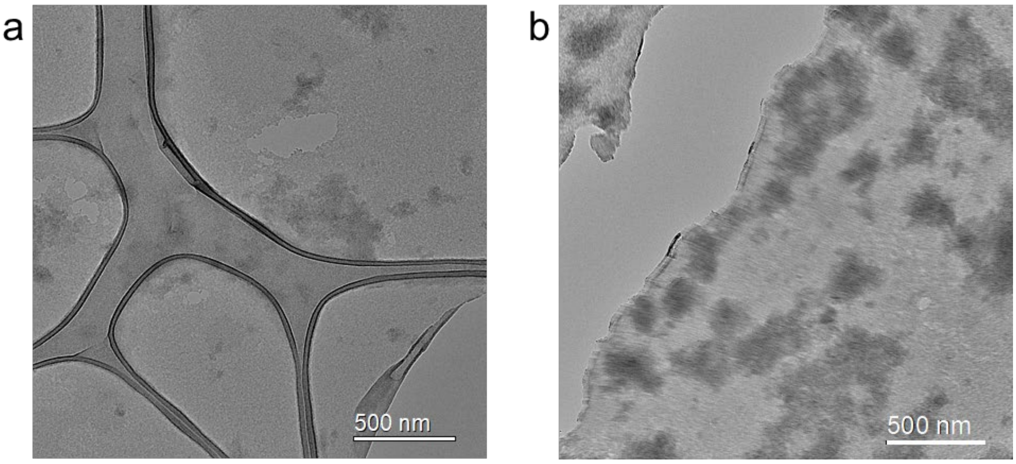

Figure S4. TEM images of assembled HHTP films guided by DHAB (a) and HDA (b).

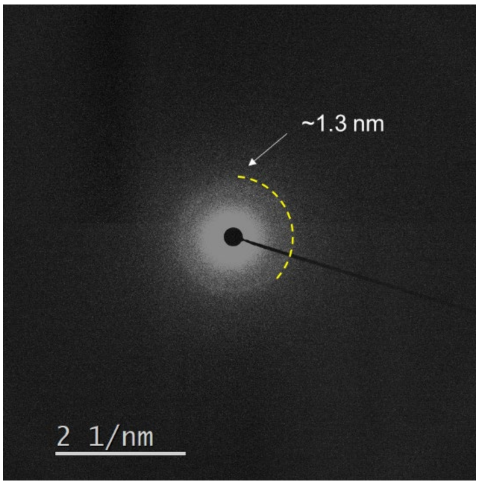

Figure S5. SAED pattern of the assembled HHTP film formed beneath the DHAB monolayer. The diffraction spacing of ~1.3 nm corresponds to the preferential face-on orientation of HHTP Notably, the face-on–oriented HHTP assembly is beam-sensitive and therefore exhibits only weak diffraction signals.

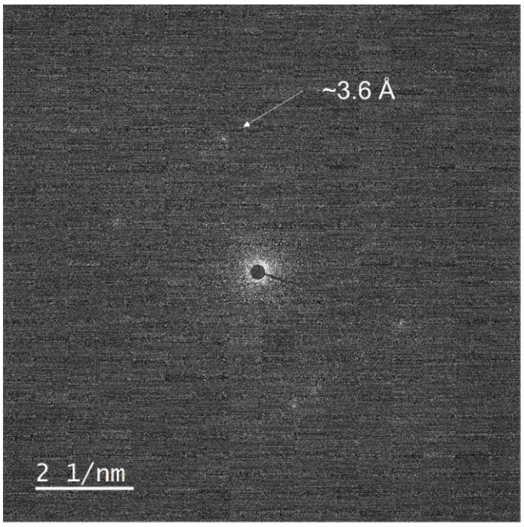

Figure S6. SAED pattern of assembled HHTP film formed beneath the HDA monolayer. The diffraction spacing of ~3.6 Å corresponds to the π–π stacking distance of the preferential edge-on orientation of HHTP.

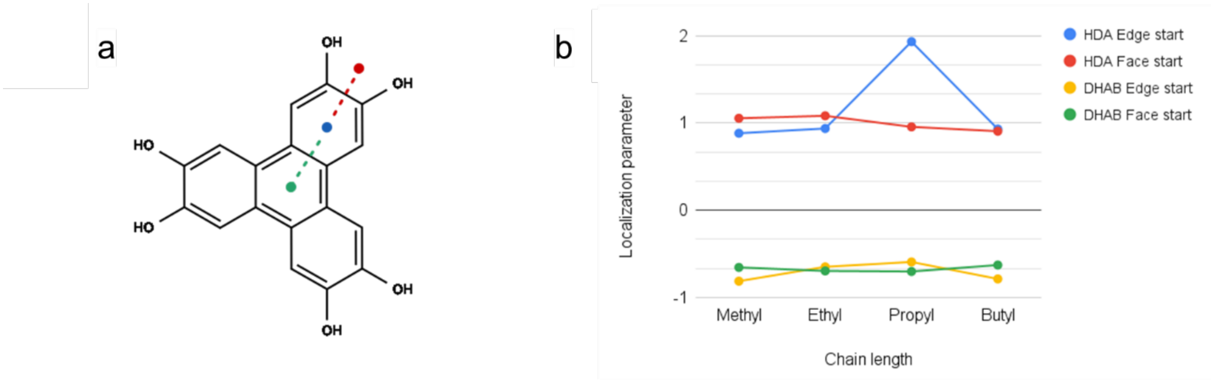

Figure S7. (a) Schematic illustration of the ligand defining three reference points to specify the surfactants' favorable position (being either at the edge (red dot) or at the central face (green dot)) or in between the blue dot. (b) The localization parameter determines whether the surfactant prefers a position close to the edge (>0) or close to the face (<0). It is calculated as the relative distance to the midpoint (blue dot). To evaluate how driving forces direct HHTP-surfactant assembly, we first calculated the interaction energies of HHTP-surfactant complexes in the gas phase in face-on and edge-on orientations. The surfactants HDA and DHAB were approximated by short-chain amine and ammonium molecules. Independent of chain length, the amine-based surfactants (approximating HDA) tend to be localized close to the hydroxyl groups, whereas the ammonium-based surfactants (approximating DHAB) tend to be localized closer to the ligand's center.

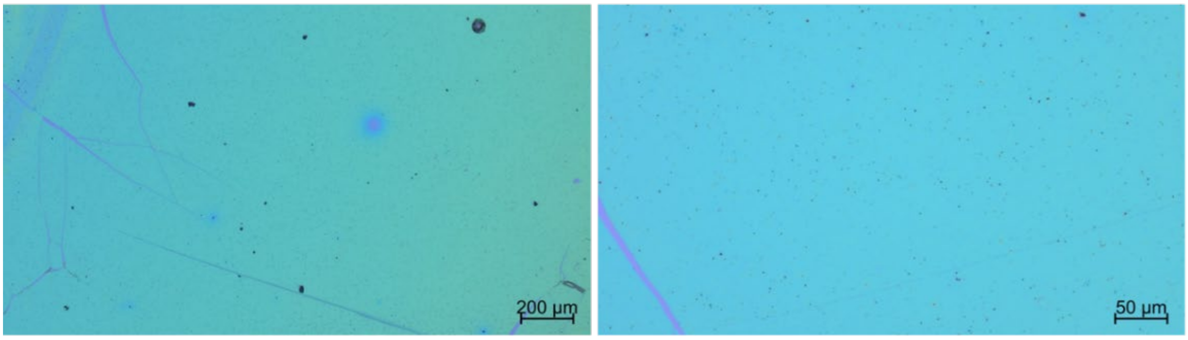

Figure S8. Optical microscopy images of the Ni-HHTP_DHAB film on the SiO_2/Si substrate.

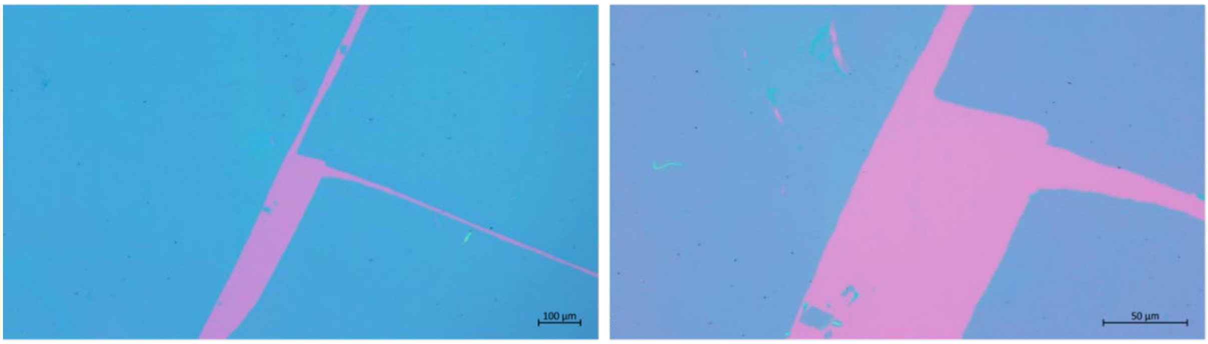

Figure S9. Optical microscopy images of the Ni-HHTP_HDA film on the SiO_2/Si substrate.

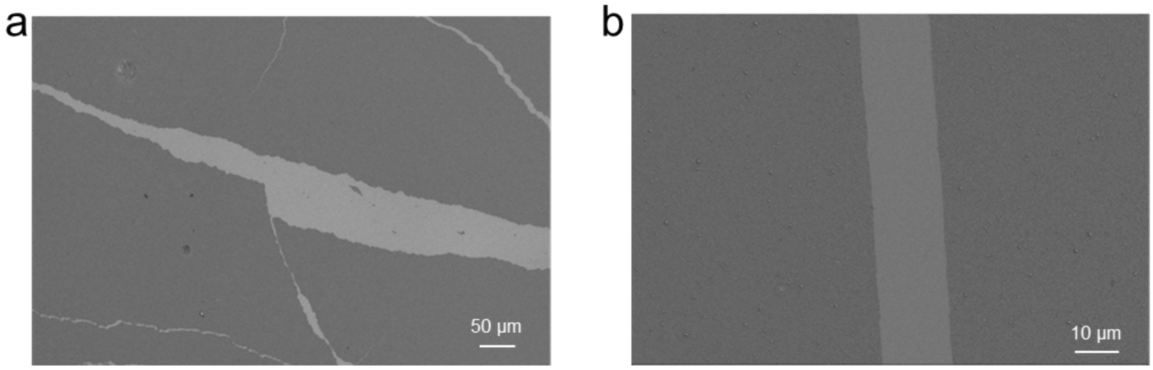

Figure S10. SEM images of the (a) Ni-HHTP_DHAB and (b) Ni-HHTP_HDA films on the Si substrate.

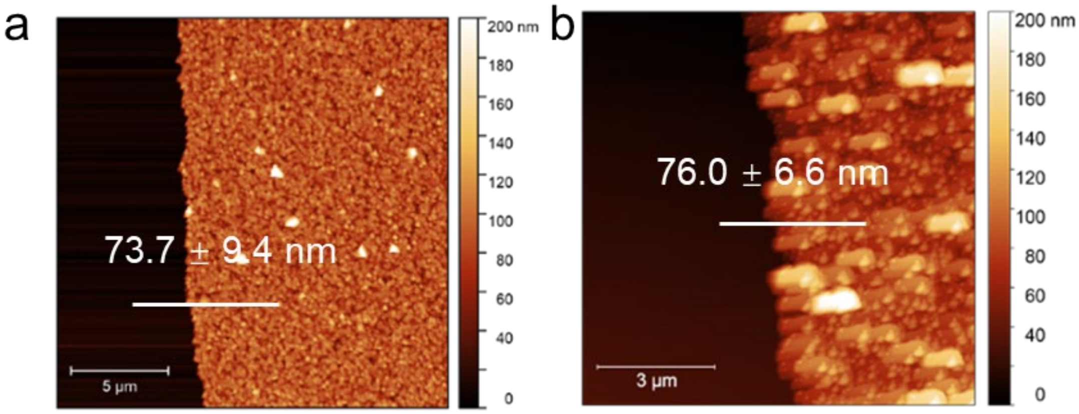

Figure S11. AFM images of the (a) Ni-HHTP_DHAB and (b) Ni-HHTP_HDA films and their averaged height.

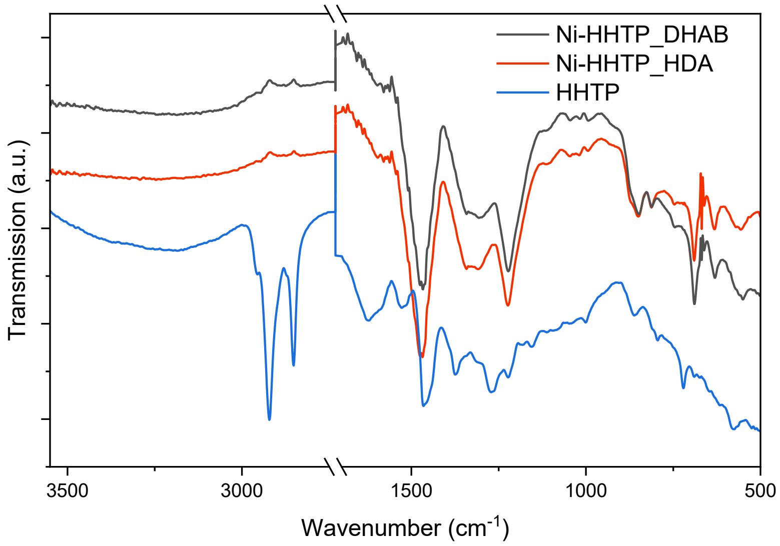

Figure S12. FTIR spectra of Ni-HHTP films and HHTP ligand.

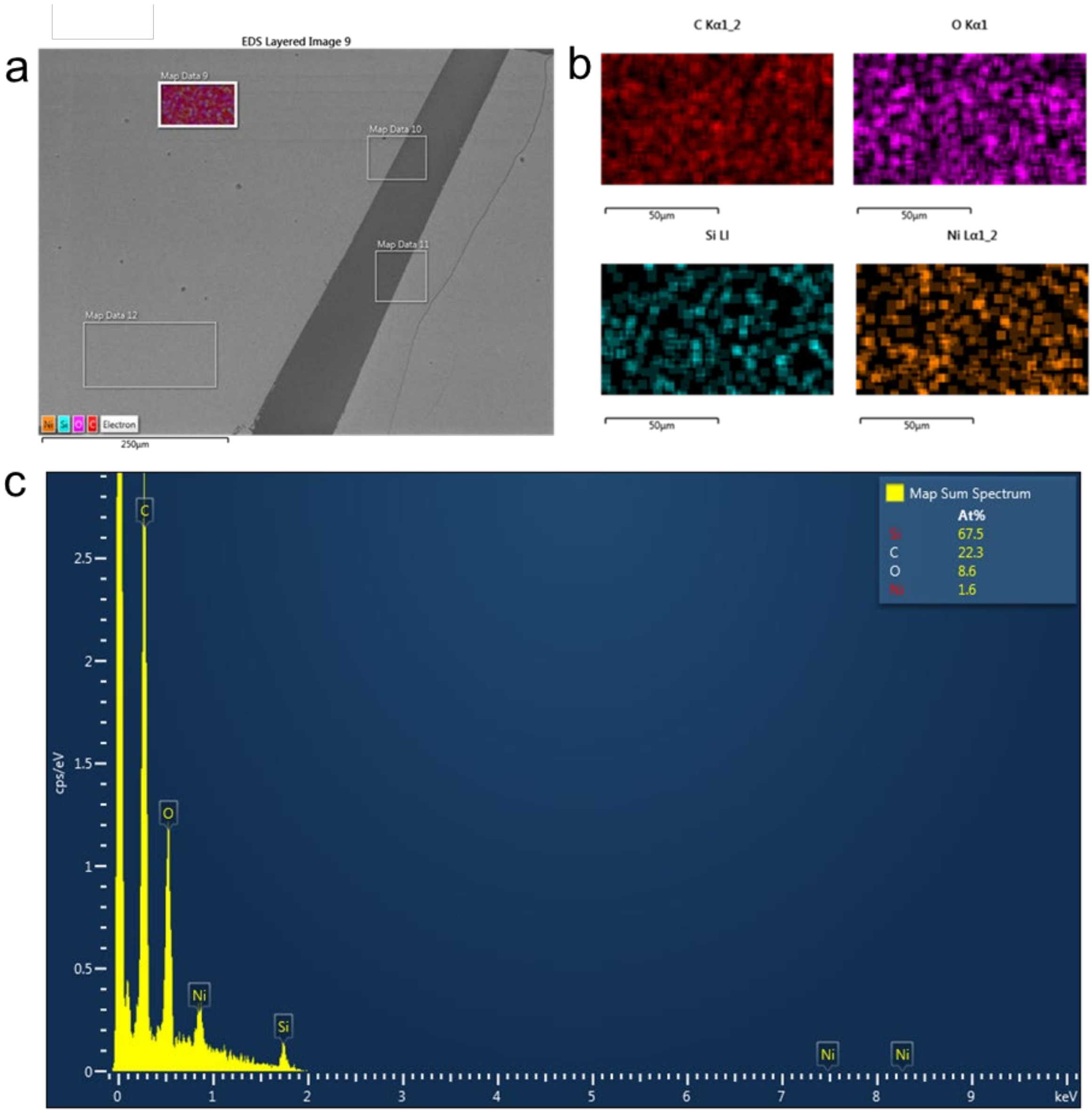

Figure S13. SEM-EDX analysis of the Ni-HHTP_DHAB film. (a) SEM image showing the selected mapping areas. (b) EDX elemental maps of C, O, Ni and Si. **c,** EDX spectrum and elemental composition of the selected region.

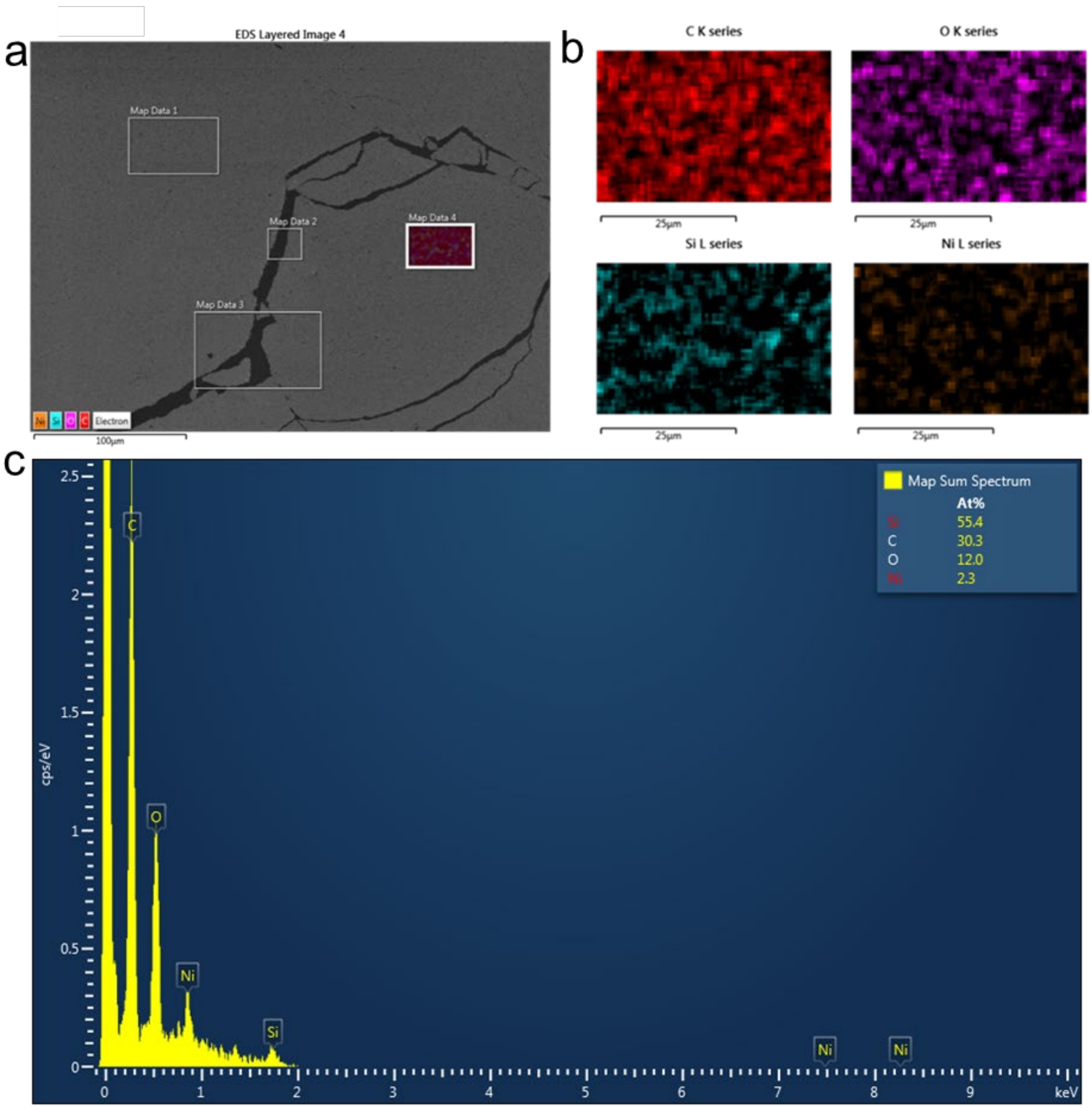

Figure S141. SEM-EDX analysis of the Ni-HHTP_HDA film. (a) SEM image showing the selected mapping areas. (b) EDX elemental maps of C, O, Ni and Si. (c) EDX spectrum and elemental composition of the selected region.

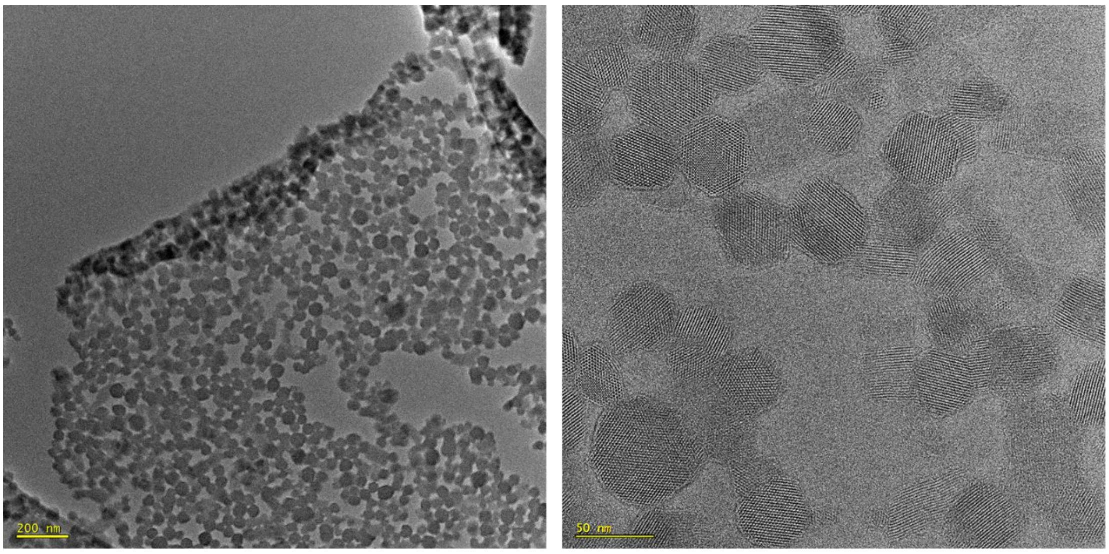

Figure S15. TEM images of the Ni-HHTP_DHAB film.

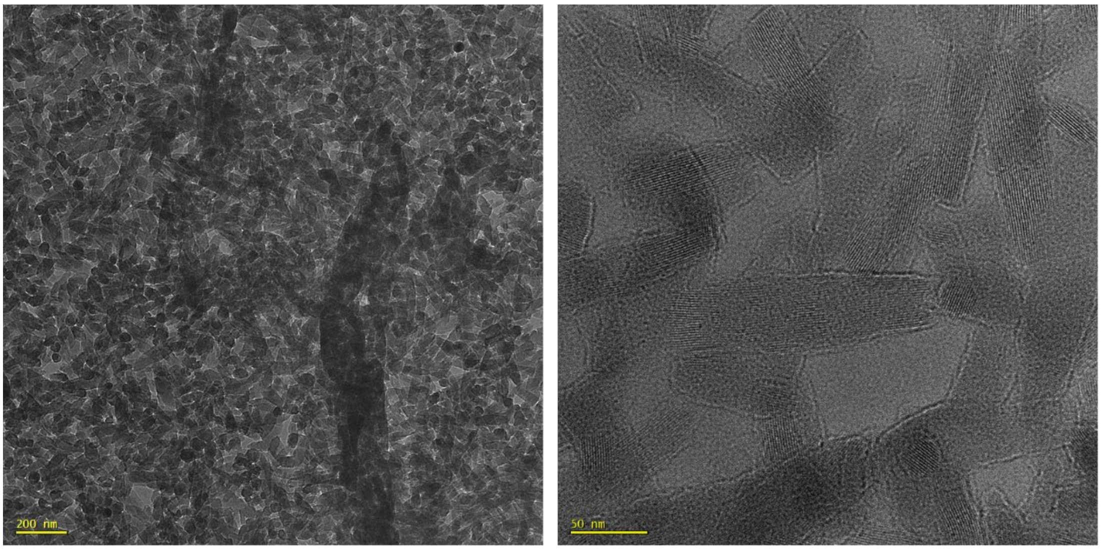

Figure S16. TEM images of the Ni-HHTP_HDA films.

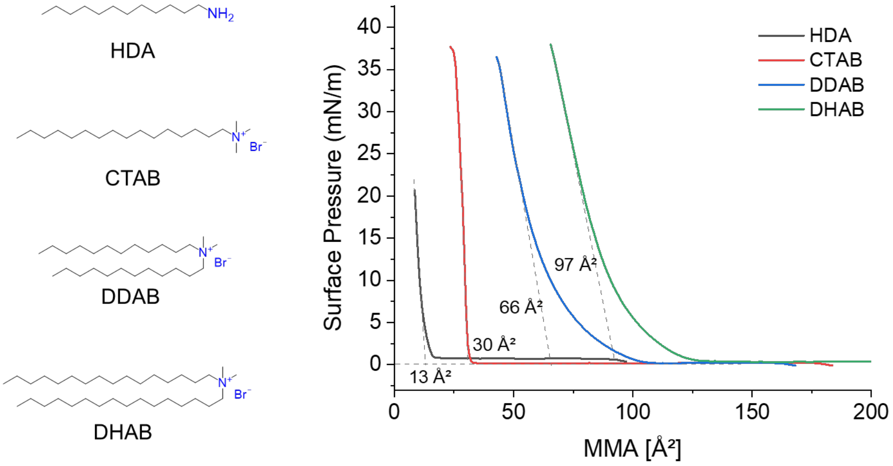

Figure S17. Chemical structures and surface pressure-area isotherms of different surfactants. To examine whether surfactant packing further affects the interfacial assembly of Ni-HHTP, we performed additional control experiments using HDA, cetyltrimethylammonium bromide (CTAB) and didodecyldimethylammonium bromide (DDAB), and DHAB (Figure S17). These surfactants differ in head-group chemistry and alkyl-chain architecture, including single-chain and double-chain structures. Surface pressure-area isotherms were measured to evaluate their monolayer packing behavior at the air-water interface. The mean molecular area (MMA) values of approximately 13, 30, 66, and 97 Å^2 molecule^{-1} for HDA, CTAB, DDAB, and DHAB, respectively. This trend suggests that longer alkyl chains and double-chain architectures increase steric effects within the monolayer, resulting in less efficient packing and a larger MMA, which correspondingly lowers the effective head-group density (Γ) (Γ_{DHAB} (1.03×10^{14} cm^{-2}) < Γ_{DDAB} (1.52×10^{14} cm^{-2}) < Γ_{CTAB} (3.33×10^{14} cm^{-2})) on the water surface. Ni-HHTP films were then synthesized under corresponding surfactant monolayers.

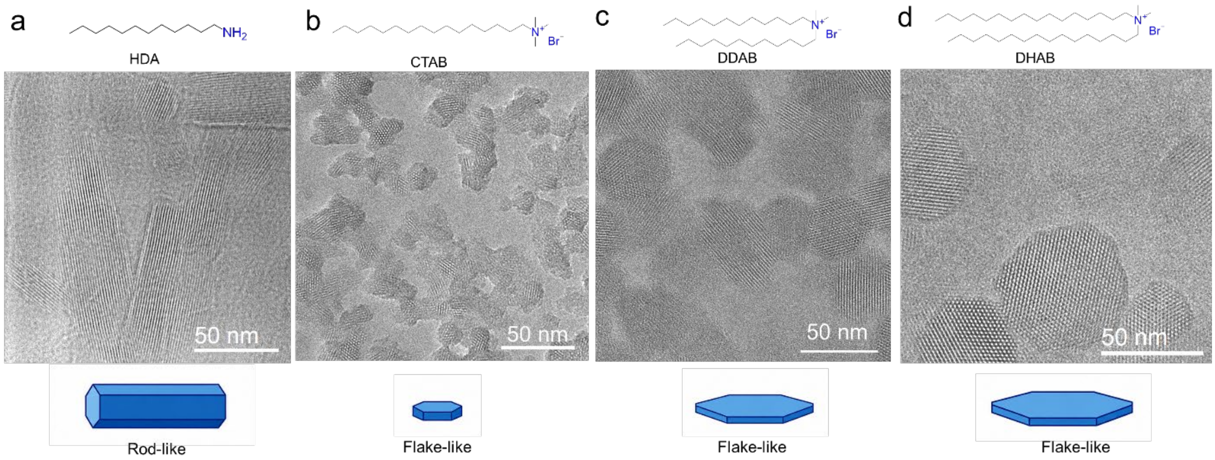

Figure S18. HRTEM of Ni-HHTP film synthesized using different surfactants. HRTEM image of (a) Ni-HHTP_HDA; (b) Ni-HHTP_CTAB; (c) Ni-HHTP_DDAB; (d) Ni-HHTP_DHAB. HRTEM analysis shows that both CTAB and DDAB systems, which share the same cationic head group as DHAB, predominantly form flake-like domains (Figure S18). However, the crystalline domain sizes of ~15 nm for Ni-HHTP_CTAB and ~45 nm for Ni-HHTP_DDAB are reduced compared with Ni-HHTP_DHAB (~55 nm), indicating that alkyl-chain architecture plays a key role in tuning nucleation and lateral growth. Overall, these results suggest that while head-group chemistry primarily determines the orientation of Ni-HHTP growth, surfactant chain architectures provide an additional handle to tune ligand preorganization on the water surface, thereby controlling nucleation density and the resulting domain size of the films.

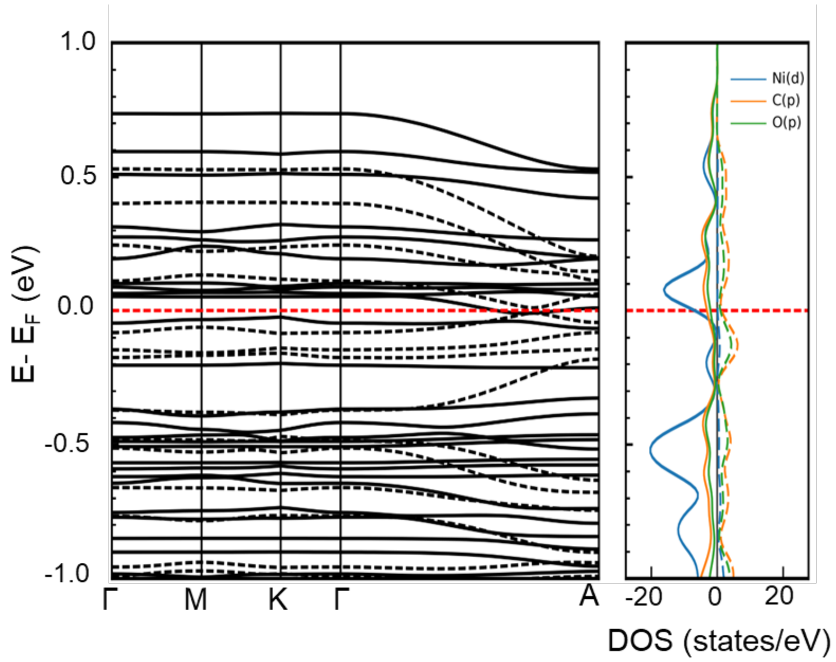

Figure S19. Calculated electronic band structure of Ni-HHTP, dash and solid lines correspond to the spin up and spin down channels, respectively. In the band structure, the in-plane K-path corresponds to Γ-M-K-Γ segments, while the out-of-plane segment is Γ-A. The calculated electronic structure and transport properties show that the system behaves as a quasi-2D layered semi-metal with strong anisotropy and pronounced spin dependence. The band structure shows both obvious band dispersion in the plane (Γ–M–K–Γ) and out of the plane (Γ–A) direction. The density of states near the Fermi level is dominated by Ni(d) states.

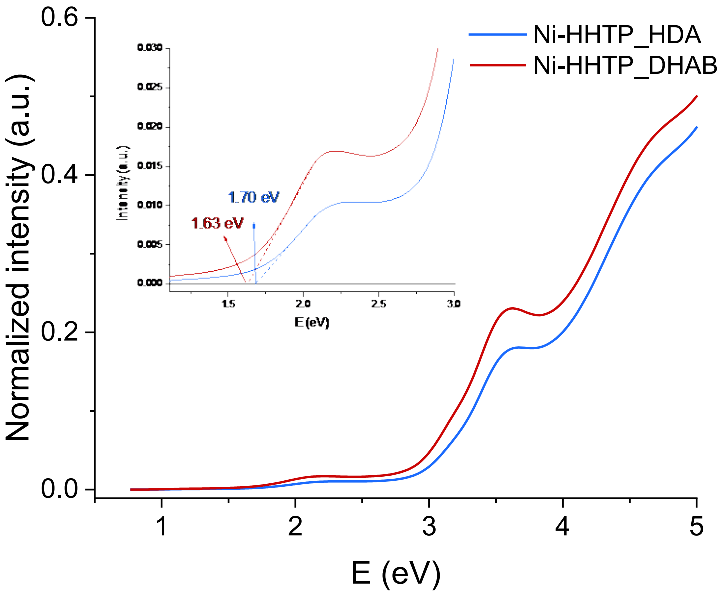

Figure S20. UV-vis absorption spectra of Ni-HHTP films. Inset: Enlarged spectra used for optical band gap calculation.

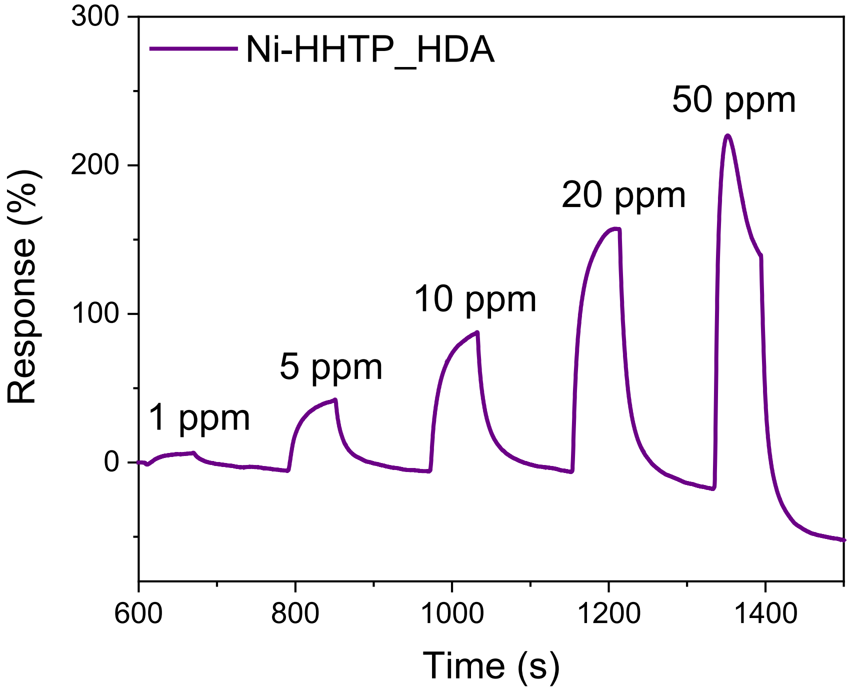

Figure S21. NH_3 sensing performances of the Ni-HHTP_HDA film at room temperature.

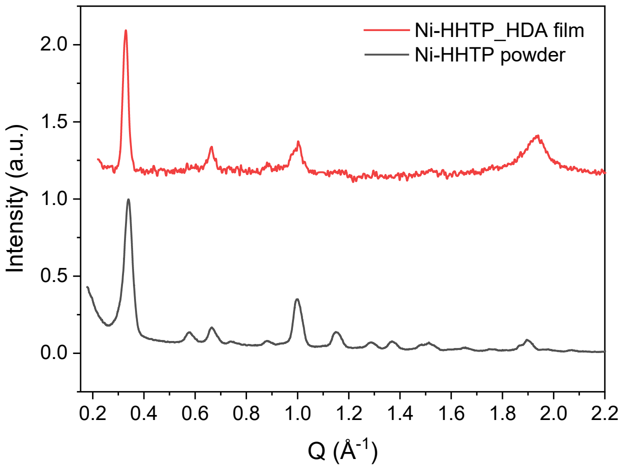

Figure S22. Comparison of the XRD pattern of Ni-HHTP powder and the GIWAXS profile of the Ni-HHTP_HDA film. The PXRD data were converted from 2θ to Q for direct comparison.

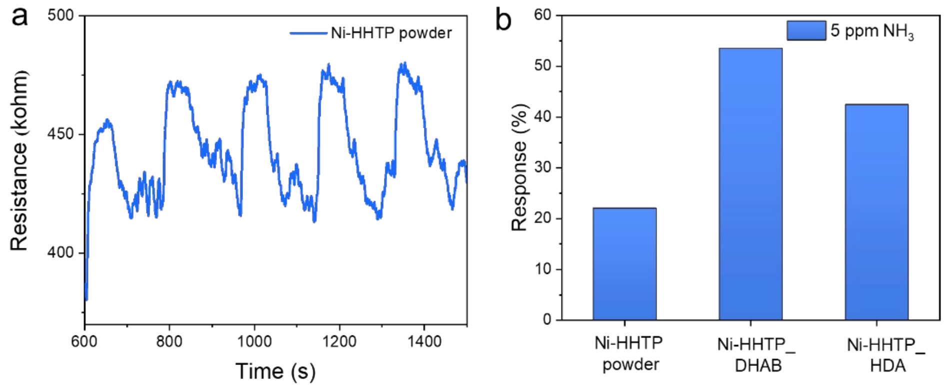

Figure S23. (a) Cycling sensing response of the Ni-HHTP powder-based device toward 5 ppm NH_3. (b) Comparison of the sensing responses of Ni-HHTP powder, Ni-HHTP_DHAB, and Ni-HHTP_HDA toward 5 ppm NH_3. To further compare the sensing performance, Ni-HHTP powder was drop-cast onto the device and tested toward 5 ppm NH_3. As shown in Figure S23a, the powder-based device shows a reproducible but relatively fluctuating response, likely due to less uniform electrical contacts in the powder layer. Its response of 22% to 5 ppm NH_3, compared with 54% for Ni-HHTP_DHAB and 42% for Ni-HHTP_HDA films (Figure S23b), suggests the advantage of continuous thin films for gas-sensing devices, underscoring the advantage of our synthetic strategy.

Table S1. NH_3 sensing performances of 2D *c*-MOF-based chemiresistive sensors at room temperature.

Materials	Response (%)	Response time (s)	LOD (ppb)	Ref
Ni-HHTP_DHAB	269.8 @50ppm	10	8.45	This work
$Cu_3(HHTP)_2$ film	129@100ppm	81.6	500	[12]
Cu-THQ	63.5@100ppm	99	20	[13]
Cu-HHTP-10C	220@100ppm	60	24	[14]
Cu-BHT	12@100ppm	58	230	[15]
Face on Cu-HHTP	262@50ppm	22.2	129	[16]
Cu-HHTP 3D film	161@100ppm	34.8	0.087	[17]
Ni/Cu-HHTP	54.6@100ppm	1174.2	1000	[18]
Cu-HHTP/Cu_2O-A	33@30ppm	63	10	[19]
Cu-HHTP@SnS_2	15.8@10ppm	79.8	9.8	[20]

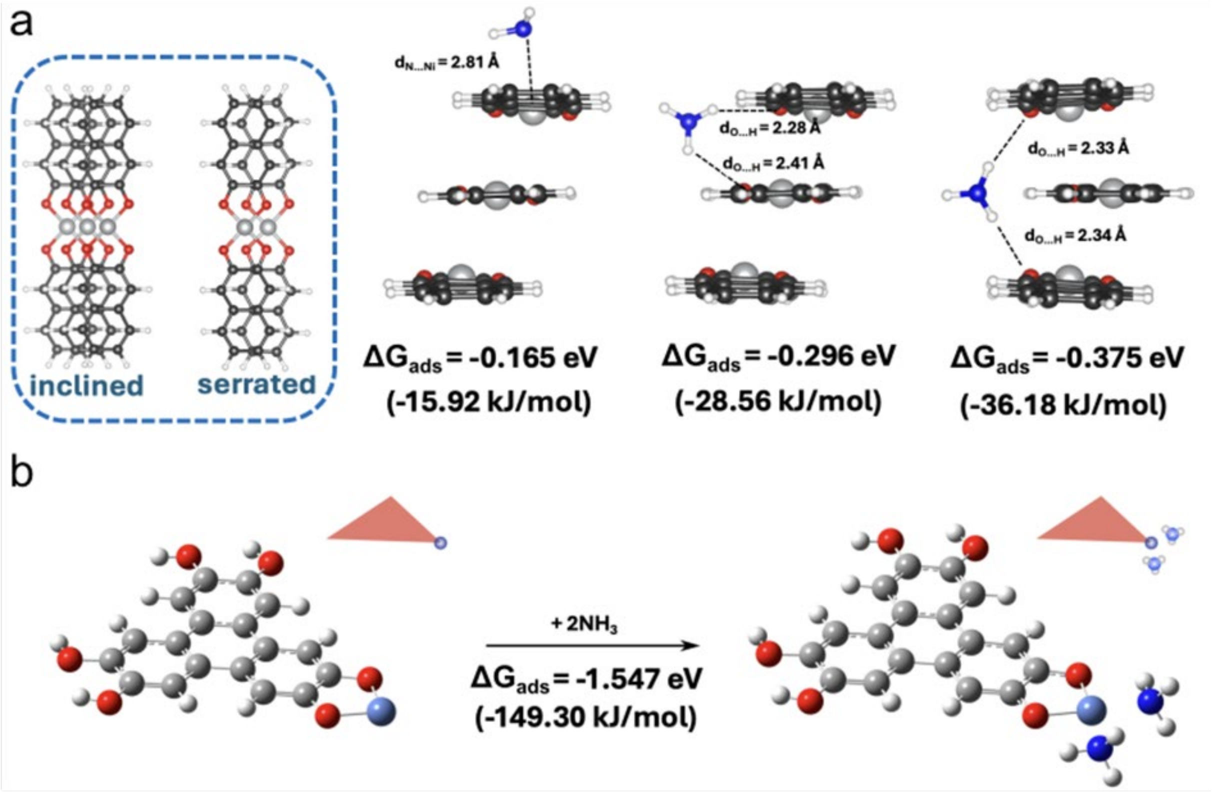

Figure S24. Computational study of NH_3 adsorption. (a) NH_3 adsorption at surface-accessible active sites and pore-accessible active sites, with inclined and serrated layer stacking configurations. (b) NH_3 adsorption at bare nickel sites. The conceivable presence of bare nickel sites, arising from possible structural defects and grain boundaries, was also identified as strongly favorable adsorption sites for NH_3.

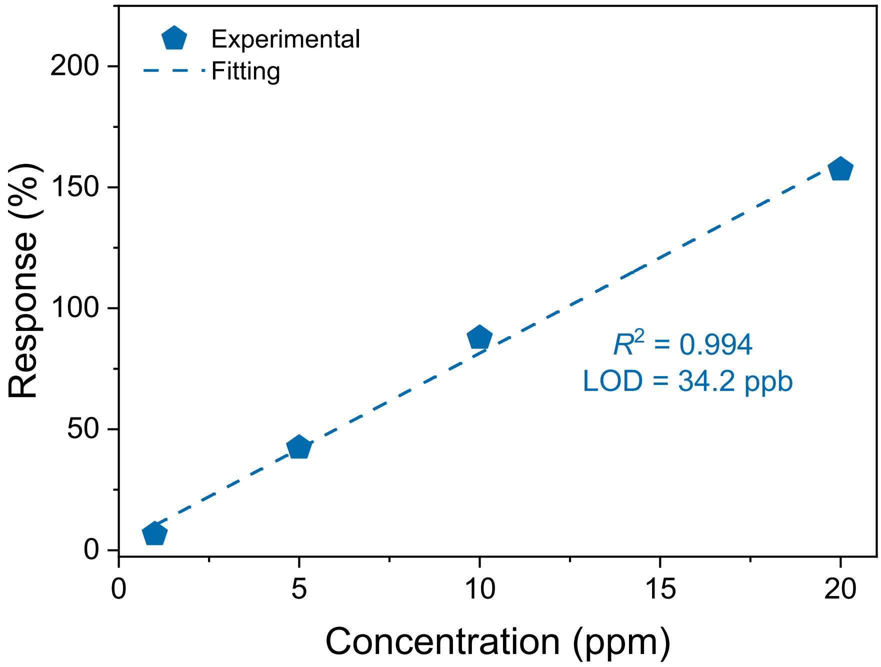

Figure S25. The linear relationship between response and NH_3 concentration of Ni-HHTP_HDA.

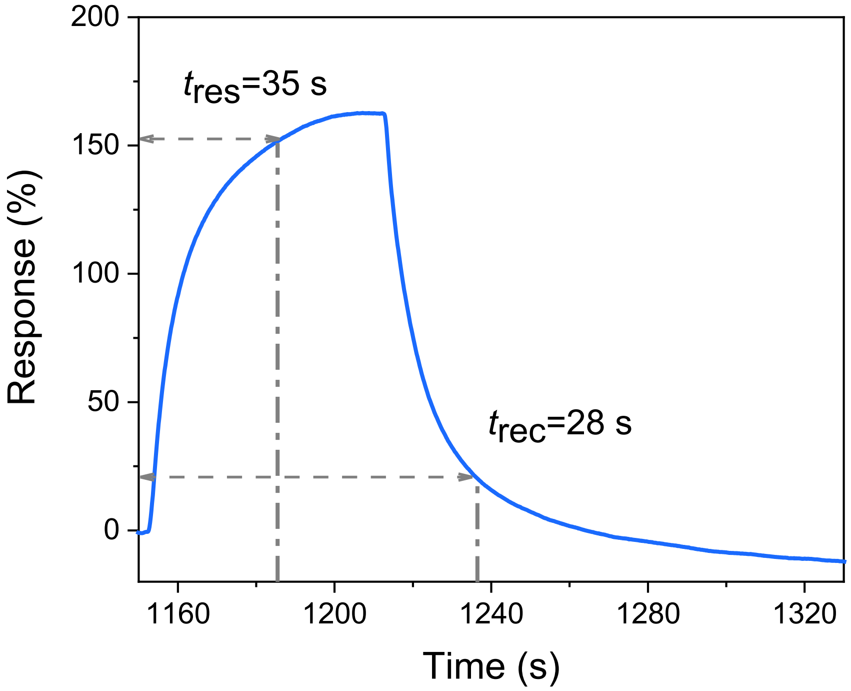

Figure S26. Response and recovery curve for Ni-HHTP_HDA@20 ppm NH_3.

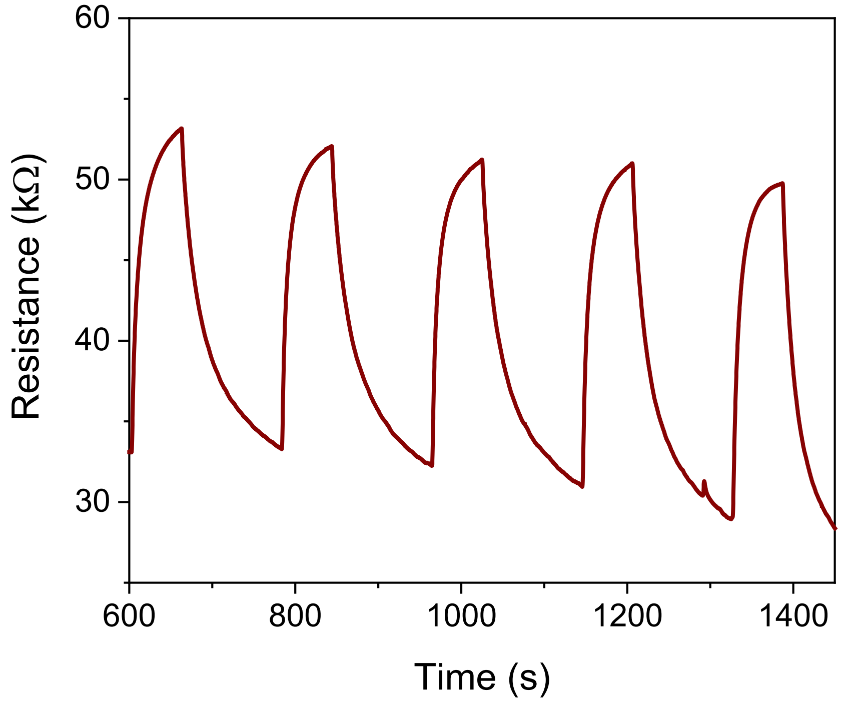

Figure S27. The cycling sensing response of Ni-HHTP_DHAB toward NH_3 (20 ppm).

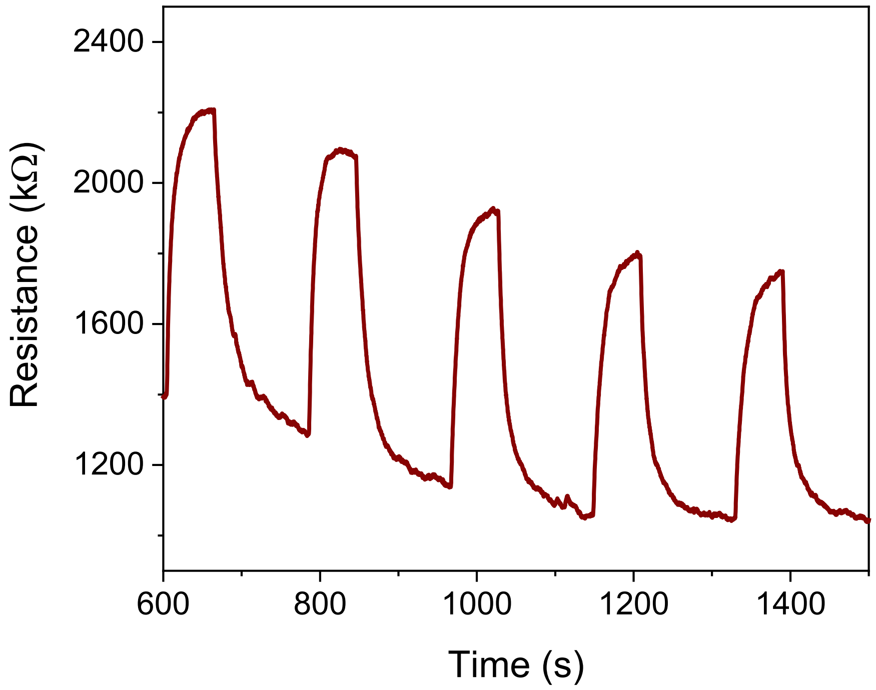

Figure S28. The cycling sensing response of Ni-HHTP_HAD toward NH_3 (20 ppm).

Reference

[1] J. P. Perdew, *Phys. Rev. Lett.* **1997**, 77, 3868.
[2] S. Grimme, J. Antony, S. Ehrlich, H. Krieg, *J. Chem. Phys.* **2010**, 132.
[3] Scm, Theoretical Chemistry, Vrije Universiteit Amsterdam, Amsterdam, The Netherlands 2025.
[4] a) G. Kresse, D. Joubert, *Phys. Rev. B* **1999**, 59, 1758; b) G. Kresse, J. Furthmüller, *Phys. Rev. B* **1996**, 54, 11169; c) G. Kresse, J. Furthmüller, *Comput. Mater. Sci.* **1996**, 6, 15.
[5] J. P. Perdew, K. Burke, M. Ernzerhof, *Phys. Rev. Lett.* **1996**, 77, 3865.
[6] S. L. Dudarev, G. A. Botton, S. Y. Savrasov, C. Humphreys, A. P. Sutton, *Phys. Rev. B* **1998**, 57, 1505.
[7] S. Grimme, S. Ehrlich, L. Goerigk, *J. Comput. Chem.* **2011**, 32, 1456.
[8] M. Hmadeh, Z. Lu, Z. Liu, F. Gándara, H. Furukawa, S. Wan, V. Augustyn, R. Chang, L. Liao, F. Zhou, E. Perre, V. Ozolins, K. Suenaga, X. Duan, B. Dunn, Y. Yamamto, O. Terasaki, O. M. Yaghi, *Chem. Mater.* **2012**, 24, 3511.
[9] V. Wang, N. Xu, J.-C. Liu, G. Tang, W.-T. Geng, *Comput. Phys. Commun.* **2021**, 267, 108033.
[10] G. K. Madsen, J. Carrete, M. J. Verstraete, *Comput. Phys. Commun.* **2018**, 231, 140.
[11] S. Fu, Universiteit Amsterdam, 2023.
[12] M. S. Yao, X. J. Lv, Z. H. Fu, W. H. Li, W. H. Deng, G. D. Wu, G. Xu, *Angew. Chem. Int. Ed.* **2017**, 56, 16510.
[13] M.-S. Yao, P. Wang, Y.-F. Gu, T. Koganezawa, H. Ashitani, Y. Kubota, Z.-M. Wang, Z.-Y. Fan, K.-i. Otake, S. Kitagawa, *Dalton Trans.* **2021**, 50, 13236.
[14] A.-Q. Wu, W.-Q. Wang, H.-B. Zhan, L.-A. Cao, X.-L. Ye, J.-J. Zheng, P. N. Kumar, K. Chiranjeevulu, W.-H. Deng, G.-E. Wang, *Nano Res.* **2021**, 14, 438.
[15] X. Chen, Y. Lu, J. Dong, L. Ma, Z. Yi, Y. Wang, L. Wang, S. Wang, Y. Zhao, J. Huang, *ACS Appl. Mater. Interfaces* **2020**, 12, 57235.
[16] M. Song, Y. Wu, J. Jia, J. Peng, Y. Ren, J. Cheng, Y. Xu, W. Liu, S. Kang, Y. Fang, *J. Am. Chem. Soc.* **2025**, 147, 17058.
[17] Y. Lin, W. H. Li, Y. Wen, G. E. Wang, X. L. Ye, G. Xu, *Angew. Chem. Int. Ed.* **2021**, 60, 25758.
[18] Y. Jiang, X. Hou, Y. Zhou, B. Wang, T. Wang, L. Zhao, J. Wei, P. Sun, G. Lu, *ACS Mater. Lett.* **2024**, 7, 76.
[19] H. Zhang, Z. Yan, Y. Ying, F. Zheng, R. Song, Z. Tang, Y. Liu, *J. Environ. Chem. Eng.* **2024**, 12, 114391.
[20] Y. Huang, X. Zhang, S. Liu, R. Wang, J. Guo, Y. Chen, X. Ma, *Chem. Eng. J.* **2023**, 458, 141364.
